# SETDB1 is critically required for uveal melanoma growth and represents a promising therapeutic target

**DOI:** 10.1038/s41419-025-08084-z

**Published:** 2025-10-24

**Authors:** Imène Krossa, Céline Pisibon, Yann Cheli, Karine Bille, Mélanie Dalmasso, Sabah Hamadat, Chrystel Husser, Marie Irondelle, Julien Cherfils-Vicini, Frédéric Soysouvanh, Sacha Nahon-Esteve, Arnaud Martel, Sandra Lassalle, Jean-Pierre Caujolle, Célia Maschi, Stéphanie Baillif, Dan Hasson, Saul Carcamo, Andrew E. Aplin, Irwin Davidson, Emily Bernstein, Valeria Naim, Robert Ballotti, Corine Bertolotto, Thomas Strub

**Affiliations:** 1 University Côte d’Azur, Nice, France; 2 Inserm, Biology and Pathologies of melanocytes, team1, Equipe labellisée Ligue 2025 and Equipe labellisée ARC 2022, Centre Méditerranéen de Médecine Moléculaire, Nice, France; 3 CNRS UMR9019 Genome Integrity and Cancers, Université Paris-Saclay, Gustave Roussy, Villejuif, France; 4 Inovarion, Paris, 75005 France; 5 Centre National de la Recherche Scientifique (CNRS) UMR7284, Inserm U1081, Institute for Research on Cancer and Aging, Nice (IRCAN), Nice, 06107 France; 6 Department of Ophthalmology, Centre Hospitalier Universitaire of Nice, Nice, France; 7 Laboratory of Clinical and Experimental Pathology (LPCE), Biobank BB-0033-00025, Nice, France; 8 Tisch Cancer Institute Bioinformatics for Next Generation Sequencing (BiNGS) Core, Icahn School of Medicine at Mount Sinai, New York, NY 10029 USA; 9 Department of Pharmacology, Physiology and Cancer Biology, Thomas Jefferson University, Philadelphia, PA 19107 USA; 10 Sidney Kimmel Comprehensive Cancer Center, Thomas Jefferson University, Philadelphia, PA 19107 USA; 11 IGBMC, CNRS UMR7104, INSERM U1258, Université de Strasbourg, Illkirch, France; 12 Department of Oncological Sciences, Tisch Cancer Institute, Icahn School of Medicine at Mount Sinai, One Gustave L. Levy Place, New York, NY 10029 USA

**Keywords:** Eye cancer, DNA replication

## Abstract

Metastatic uveal melanomas are highly resistant to all existing treatments. To identify actionable vulnerabilities, we conducted a CRISPR-Cas9 knockout screen using a library composed of chromatin regulators. We revealed that the lysine methyltransferase, SETDB1, plays a critical role in metastatic uveal melanoma cell proliferation and survival. Functionally, SETDB1 deficiency induces a DNA damage response, senescence-like state and growth arrest. Knockdown of SETDB1 is associated with a decreased expression of genes related to replication and cell cycle. Moreover, deficiency in CDC6, an essential regulator of DNA replication, phenocopies SETDB1 inhibition. Using a pre-clinical model, we further demonstrated that anti-SETDB1 therapy impairs tumor growth in vivo. Therefore, we not only provide evidence that SETDB1 plays a critical role in metastatic uveal melanoma cell growth, but we also identify SETDB1 as a novel relevant therapeutic target for the treatment of metastatic uveal melanoma.

## Introduction

Uveal melanoma is the most common primary intraocular malignancy in adults and a deadly neoplasm. Despite successful treatment of the primary lesion by proton therapy or enucleation, up to 50% of uveal melanoma patients develop metastases, predominantly in the liver (reviewed in [1]). Metastatic uveal melanomas are highly refractory to existing treatments. Recently, tebentafusp (Kimmtrak), a bispecific protein immunotherapy targeting CD3 and the melanoma antigen GP100, has been shown to improve the overall survival of patients with metastatic uveal melanoma [2]. However, tebentafusp treatment is limited to patients with an HLA-A*02:01 haplotype and demonstrated benefit in a small subset of them [3]. To date, ninety percent of patients with metastatic uveal melanoma die within 6 months after diagnosis of metastases, highlighting an unmet clinical need including HLA-independent strategies. The characterization of novel oncogenic molecular mechanisms driving uveal melanoma progression and treatment resistance is essential to improve patients’ survival.

The main oncogenic drivers in uveal melanomas are mutations in the heterotrimeric G-protein alpha subunit GNAQ or its paralog GNA11 (GNAQ/11). Ninety percent of uveal melanomas harbor a mutation in one of these two genes [4]. The most frequent GNAQ and GNA11 mutation is the substitution of glutamate at position 209 by proline or leucine (GNAQ/11^Q209P/L^) that results in loss of GTPase activity, producing constitutive activation of *GNAQ/GNA11*. Over the past decade, studies dissecting the molecular mechanisms of uveal melanoma progression have revealed that mutant GNAQ/11 signals through activation of broad downstream signaling modules, including PLCβ-PKC, MEK-ERK, and Hippo-YAP (reviewed in [1]), making GNAQ/11 and/or their downstream pathways attractive targets for anti-uveal melanoma therapies. While drugs targeting these pathways, either alone or in combination, impair uveal melanoma cell growth in vitro, clinical trials have showed limited, if any, efficacy [5]. Thus, advances in the molecular characterization of uveal melanoma over these years have not yet translated into effective therapeutic strategies to prevent or eliminate metastasis. Hence, it remains essential to identify pivotal players in metastatic uveal melanoma proliferation and survival that would be amenable to therapeutic intervention.

Complementary to *GNAQ/11* driver mutations, uveal melanoma is characterized by secondary alterations, the most frequent is the loss of the tumor suppressor BRCA1-associated protein-1 (*BAP1*) gene. BAP1 loss is associated with a high metastatic risk and a poor prognosis [4, 6]. BAP1 is a deubiquitinase with a substrate preference for histone H2A lysine 119 (H2AK119), meaning that BAP1 loss triggers accumulation of H2AK119 mono-ubiquitination, which in turn promotes transcriptional repression [7]. Accumulating evidence indicate that epigenetic changes play important role in cancer progression, but also therapy resistance [8]. Little is known about the mechanisms of epigenetic regulation in uveal melanoma cell biology. A few key roles have been identified for histone deacetylases (HDAC), such as HDAC2 or HDAC4 [9–11], lysine methyltransferases [12, 13], or chromatin remodeling complexes [14, 15] in the pathogenesis of uveal melanoma. While these molecules emerged as promising drug targets in uveal melanoma treatment, none, including the well-known pan-HDAC inhibitors (e.g., vorinostat, entinostat), tested so far showed clinical efficacy [8]. Thus, uncovering epigenetic regulators that can be targeted therapeutically in uveal melanoma is of critical importance.

In order to probe the role of epigenetic-related mechanisms involved in uveal melanoma proliferation and survival, in this study, we performed a CRISPR-Cas9 screen in GNAQ^Q209P^ human uveal melanoma cells targeting chromatin modifiers with or without enzymatic activities. We identified the lysine methyltransferase SETDB1, thereby providing the first evidence of SETDB1 implication in uveal melanoma cell proliferation and survival.

## Results

### A CRISPR-Cas9 screen identifies SETDB1 as a key driver of metastatic uveal melanoma cell growth

To identify actionable vulnerabilities in metastatic uveal melanoma cells, we performed a CRISPR-Cas9 knockout screen targeting ~140 chromatin remodelers with or without enzymatic activity in GNAQ^Q209P^ uveal human melanoma cells [16]. Briefly, OMM1.3 uveal melanoma cells, originally derived from liver metastasis and harboring a GNAQ^Q209P^ mutation, were engineered to stably express Cas9, transduced with GFP-tagged single-guide RNA (sgRNA) library (3–4 sgRNAs per gene encoded in pLKO.1) and GFP-positive cells were sorted for expansion.

**Fig. 1:**
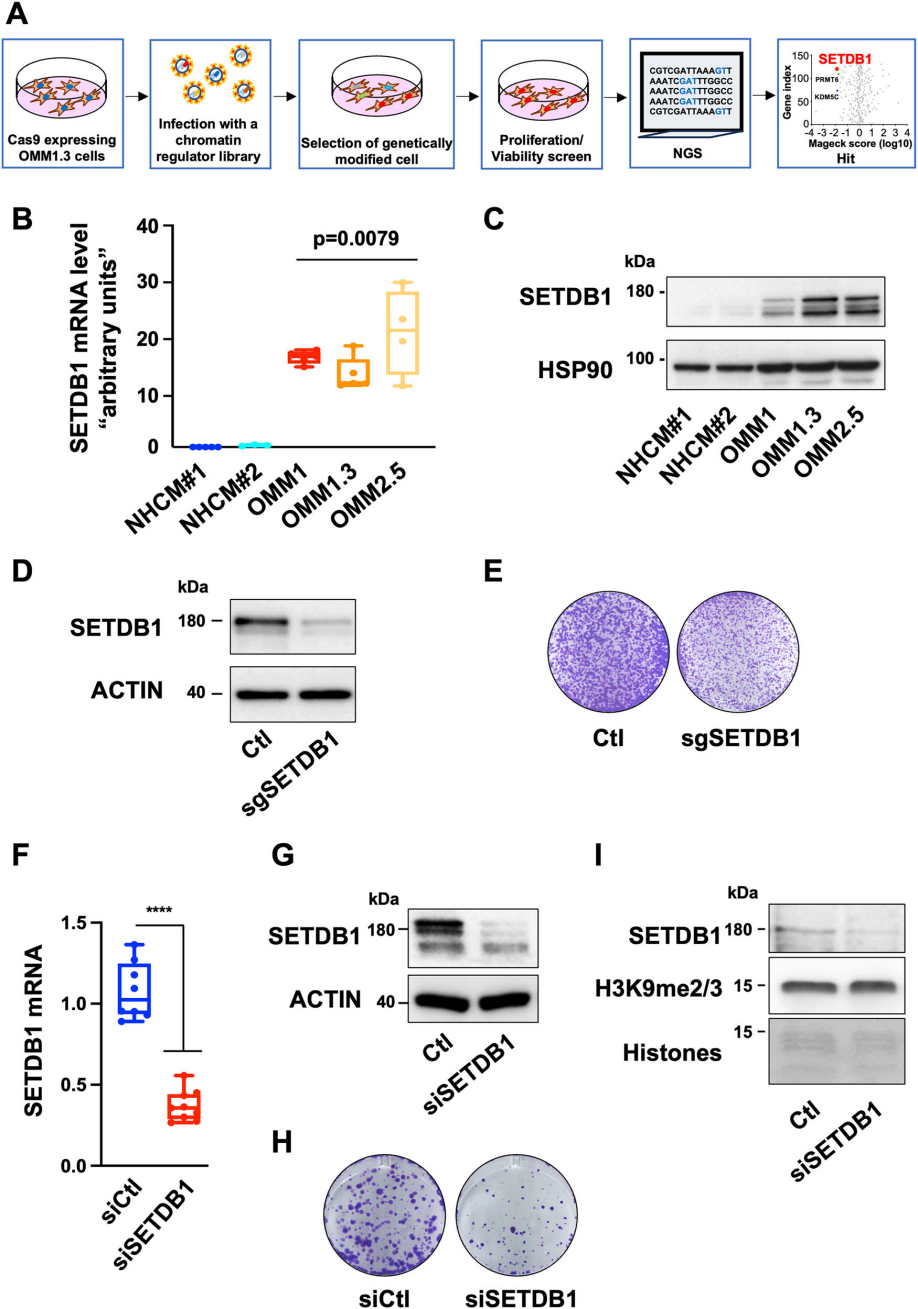
SETDB1 is critically required for metastatic uveal melanoma cell growth. **A** Schematic of the CRISPR-Cas9 chromatin-regulators screen with Log_10_-transformed MAGeCK robust ranking aggregation (RRA)-scores for either depletion (left) or enrichment (right) of sgRNAs in OMM1.3 cells at D35 compared to D0. **B** RT-qPCR analysis of SETDB1 in metastatic uveal melanoma cells (OMM1, OMM1.3, OMM2.5) compared to normal human uveal melanocytes from two different patients (NHCM#1 and NHCM#2). Mann–Whitney test was performed for comparison between groups, *n* = 9. Data are the mean ± SEM. **p = 0.0079. **C** Immunoblot analysis of SETDB1 in metastatic uveal melanoma cells compared to normal human choroidal melanocytes. HSP90 was used as a loading control. **D** Immunoblot of SETDB1 in the indicated whole-cell lysates of OMM1.3 infected with control sgRNA (Ctl) or a sgRNA to SETDB1 cell lines. β-Actin was used as a loading control. **E** OMM1.3 Ctl and pooled SETDB1-KD cells were seeded at the same density and cultured for 10 days. Representative images of three independent experiments are shown. **F** Representative box and whiskers plots of RT-qPCR analysis of SETDB1 in OMM1.3 cells treated with control siRNA (siCtl) or an siRNA to SETDB1 (siSETDB1) for 48 h. Mann–Whitney test was performed for comparison between groups, *n* = 5. Data are mean ± SEM. ****p < 0.0001. **G** Immunoblot analysis to SETDB1 in the indicated whole cell lysates of OMM1.3 cells treated for 6 days. β-Actin was used as a loading control. **H** OMM1.3 cells treated as in (**F**) were seeded at the same density and cultured for 10 days. Representative images of three independent experiments are shown. **I** Immunoblot of SETDB1 and H3K9me2/3 in the indicated chromatin fraction of OMM1.3 cell line (96 h). Histones were used as a loading control.

Next, genomic DNA was isolated from cells at day 0, which represents the library distribution prior to the screening selection process, and at day 35, and the abundance of each sgRNA was determined using next-generation sequencing. Analysis of the CRISPR-Cas9 screen dataset with MaGeck software, which calculates a score based on a fold change, revealed depleted (left part of the volcano plot) or enriched (right part of the volcano plot) sgRNA compared to the control condition (Fig. 1A).

To identify essential genes involved in cellular proliferation and survival, we have used a “dropout” strategy in which cells with the phenotype of interest are depleted in the screen. This approach is reflected by the number of sgRNAs against these genes being strongly decreased from the population, whereas other sgRNAs are maintained. By focusing our attention on genes whose loss conferred a reduced growth advantage on cells the screen yielded several candidates, with enrichment of factors that mediate histone methylation among the top 10 hits (Table 1). These include the histone demethylases KDM5C and KDM4D, as well as the histone methyltransferases PRMT6 and SETD1B. The lysine methyltransferase SETDB1 [17], that catalyzes the addition of methyl groups to histone H3 at lysine 9 (H3K9) and non-histone proteins, was the most valuable hit using the MaGeck software (Fig. 1A).

**Table 1:** Top 10 hits from the CRISPR-Cas9 screen using MAGeCK robust ranking aggregation (RRA)-scores of sgRNAs in OMM1.3 cells at D35 compared to D0.

id	num	neg|score	neg|*p*-value	neg|fdr	neg|rank	neg|goodsgrna	neg|lfc	pos|score	pos|*p*-value	pos|fdr	pos|rank	pos|goodsgrna	pos|lfc
DAXX	4	0.00023702	0.0010823	0.152602	1	3	−0.93234	0.12217	0.20515	0.923331	26	1	−0.93234
SETDB1	4	0.013236	0.035616	0.782619	2	2	−0.39488	0.71051	0.70898	0.974234	100	0	−0.39488
KDM5C	4	0.014218	0.03783	0.782619	3	2	−0.18554	0.13538	0.22226	0.923331	30	1	−0.18554
PRMT6	4	0.01819	0.046837	0.782619	4	2	−0.37276	0.69276	0.6914	0.974234	99	0	−0.37276
CHD1L	4	0.025396	0.063406	0.782619	5	1	−0.53367	0.71908	0.71756	0.974234	102	0	−0.53367
MLLT11	2	0.027284	0.04986	0.782619	6	1	−0.38805	0.21259	0.22753	0.923331	44	1	−0.38805
KDM4D	4	0.029838	0.073351	0.782619	7	2	−0.23637	0.46253	0.49102	0.958623	68	1	−0.23637
PRDM14	4	0.030329	0.074418	0.782619	8	3	−0.11042	0.29956	0.38445	0.958623	54	1	−0.11042
HIRA	4	0.032562	0.079555	0.782619	9	1	−0.2559	0.65485	0.65356	0.964831	95	0	−0.2559
SETD1B	4	0.039688	0.094747	0.782619	10	2	−0.1836	0.6482	0.64722	0.964831	93	1	−0.1836

We also found approximately a 15-fold higher SETDB1 mRNA level in metastatic uveal melanoma cells as compared to human uveal melanocytes (Fig. 1B), suggesting its upregulation along with tumor transformation and progression. This observation was confirmed at the protein level showing higher SETDB1 expression in metastatic uveal melanoma cells compared to normal uveal melanocytes (Fig. 1C).

Given that SETDB1 was identified as one of the top critical genes for metastatic uveal melanoma cell growth, that its expression is higher in uveal melanoma cells as compared to normal uveal melanocytes and that its role in uveal melanoma biology remained unknown, we further focused our studies on SETDB1.

The functional impact of SETDB1 inhibition on growth was validated by introducing individual sgRNAs. SETDB1 depletion in pooled OMM1.3 metastatic uveal melanoma cells was confirmed by immunoblot (Fig. 1D) and resulted in a substantially decreased cell growth (Fig. 1E). We further validated these results using an siRNA that efficiently downregulated SETDB1 at both the mRNA and protein level (Fig. 1F, G). Likewise, silencing of SETDB1 expression with siRNA resulted in reduced cell growth ability (Fig. 1H). We extended these findings in two other metastatic uveal melanoma cell lines, which harbor typical *GNAQ* (OMM2.5) or *GNA11* (OMM1) hotspot mutations (Supplementary Fig. 1A, B). Our data also showed that SETDB1 knockdown reduced the growth ability of primary uveal melanoma cells that carry a *GNAQ* mutation and do not express BAP1 (MP46) (Supplementary Fig. 1C). It is noteworthy that an efficient decrease of SETDB1 was observed after 48 h while the growth was affected at a later time, indicating that SETDB1 is a mechanistic driver of growth arrest rather than a downstream consequence. Given that SETDB1 is a histone lysine methyltransferase that mainly catalyses H3K9 di- and tri-methylation [17], we assessed the level of H3K9 di- and tri-methylated histone marks. Unexpectedly, SETDB1 knockdown did not result in global changes in H3K9 di- and tri-methylation, suggesting other compensatory mechanisms (Fig. 1I). Collectively, our data indicate that SETDB1 plays a critical role in metastatic uveal melanoma cell growth but likely acts independently of H3K9 di- and tri-methylation alterations.

### SETDB1 regulates DNA replication and genomic integrity

**Fig. 2:**
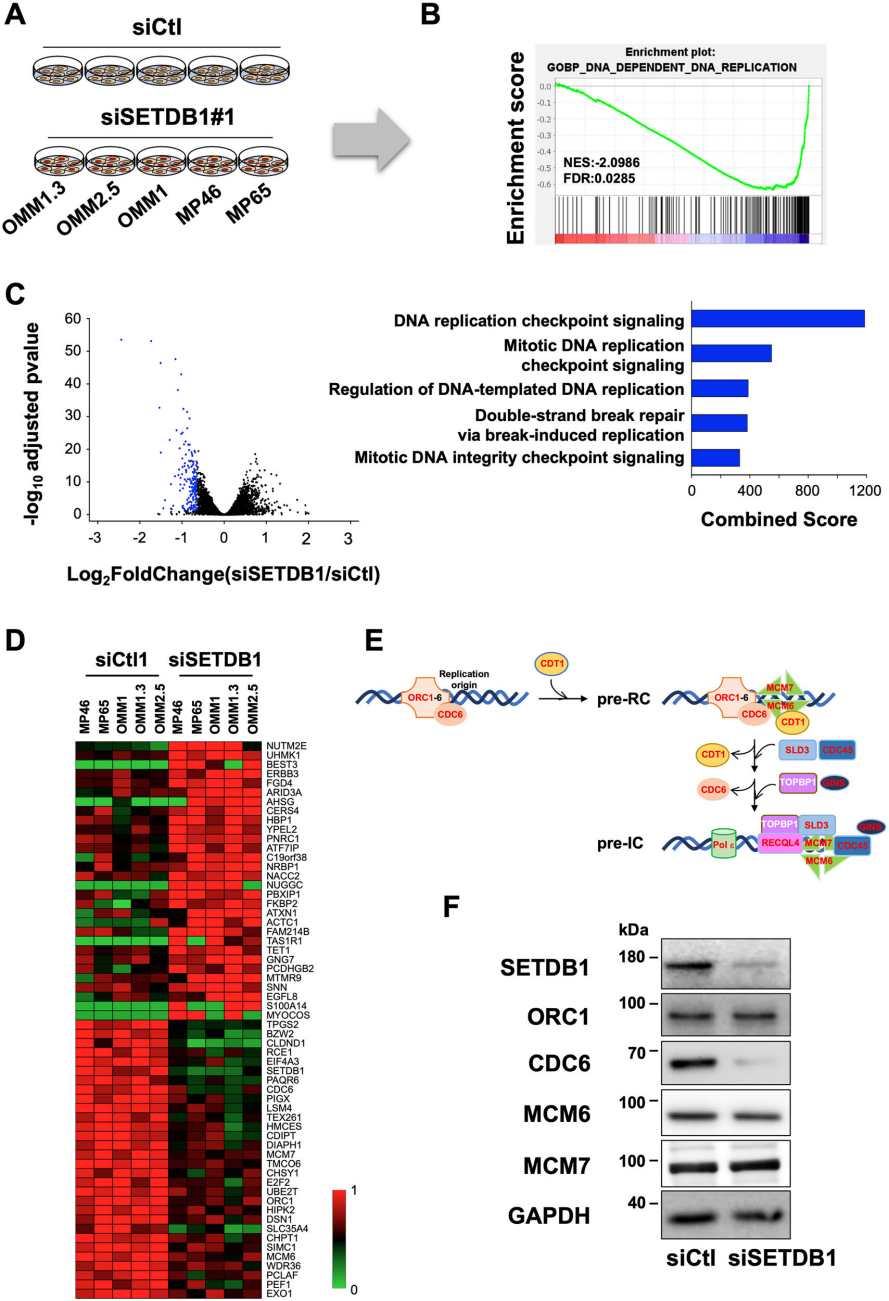
SETDB1 regulates DNA replication. **A** Schematic representation of the experimental design. **B** GSEA analysis from OMM1.3 siSETDB1 cells versus OMM1.3 siCtl cells, data queried against the “GOBP” group of gene sets of all genes in the 5 different cell lines upon 48 h of siSETDB1 over siCtl. **C** Volcano-plot representing -log10(adjusted *p*-value) as a function of the estimated log2 Fold-Change of all genes in the 5 different cell lines upon 48 h of siSETDB1 over siCtl. Significantly downregulated genes (179 genes) are shown in blue (left). Functional annotation (biological processes) of the 179 genes commonly downregulated after 48 h of siSETDB1 over siCtl in the 5 different cell lines. Categories are ranked by combined score (right). **D** Heat Map of the top 60 deregulated genes representing relative expression. **E** Schematic representation of the pre-initiation complex. **F** Immunoblot of the indicated proteins in whole-cell lysates of OMM1.3 cells treated with a control (siCtl) or SETDB1 siRNA for 48 h. GAPDH was used as a loading control.

To delineate the mechanisms by which SETDB1 regulates metastatic uveal melanoma cell growth, we profiled the transcriptome of five different uveal melanoma cell lines. Given the molecular distinctions driving the clinical outcome in uveal melanoma, we included both BAP1-positive and BAP1-negative cell lines to ensure that our findings would not be limited to specific BAP1 status and represent a broader spectrum of the disease. Therefore, we have used 3 metastatic BAP1 positive cell lines (OMM1.3, OMM2.5 and OMM1) and 2 primary BAP1 negative cell lines (MP46 and MP65) that were either treated with a control siRNA or with an siRNA directed against SETDB1 (Fig. 2A and Supplementary Fig. 2A). Gene Set Enrichment Analysis (GSEA) uncovered 4 gene sets (out of 10 statistically significant) related to DNA replication, and others related to telomere and cell cycle in SETDB1-KD cells compared to control cells (Fig. 2B and Supplementary Fig. 2B). Moreover, our transcriptomic analyses identified a set of common genes across the five SETDB1-KD cell lines that were significantly downregulated (*n* = 179) (Table 2) compared to control cells with enrichment of DNA replication biological processes (Fig. 2C). Supporting this, the heatmap revealed several genes implicated in DNA replication origin licensing and assembly of pre-replication complex (ORC1, CDC6, MCM6, MCM7) and cell cycle (E2F2) (Fig. 2D, E). It is worth noting that CDC6 is regulated by E2F proteins [18]. RNA-seq datasets also revealed reduced mRNA level of other components of the pre-initiation complex (CDT1, CDC45, and SLD3/TICRR) in SETDB1-KD cells compared to control cells. Reduction of CDC6 and MCM6 in SETDB1 KD OMM1.3 cells was confirmed at the protein level (Fig. 2F). These data suggest that SETDB1 plays a critical role in uveal melanoma cell growth through regulation of DNA replication.

**Table 2:** Common genes across the five SETDB1-KD cell lines that were significantly downregulated compared to control cells.

Ensembl Gene ID	Gene name	Description	Log2 FC (siSETDB1 vs siLuc)	Adjusted *p*-value (siSETDB1 vs siLuc)
ENSG00000080822	CLDND1	Claudin domain containing 1	−2.44067942	2.58731E-54
ENSG00000176087	SLC35A4	Solute carrier family 35 member A4	−1.730216179	6.70621E-54
ENSG00000136261	BZW2	Basic leucine zipper and W2 domains 2	−1.53357307	1.77831E-33
ENSG00000143379	SETDB1	SET domain bifurcated histone lysine methyltransferase 1	−1.507300673	3.46712E-47
ENSG00000176720	BOK	BCL2 family apoptosis regulator BOK	−1.503384978	1.02184E-19
ENSG00000197415	VEPH1	Ventricular zone expressed PH domain containing 1	−1.440234166	0.011404577
ENSG00000126970	ZC4H2	Zinc finger C4H2-type containing	−1.402321242	3.63752E-05
ENSG00000108774	RAB5C	RAB5C, member RAS oncogene family	−1.288855293	1.31662E-23
ENSG00000135678	CPM	Carboxypeptidase M	−1.260576418	4.44947E-10
ENSG00000100191	SLC5A4	Solute carrier family 5 member 4	−1.259885791	0.014637052
ENSG00000101282	RSPO4	R-spondin 4	−1.24160913	0.021345207
ENSG00000236609	ZNF853	Zinc finger protein 853	−1.208217162	0.001938456
ENSG00000168061	SAC3D1	SAC3 domain containing 1	−1.163129084	1.60468E-12
ENSG00000112576	CCND3	Cyclin D3	−1.152456908	2.45482E-48
ENSG00000134779	TPGS2	Tubulin polyglutamylase complex subunit 2	−1.129447327	1.53548E-26
ENSG00000163870	TPRA1	Transmembrane protein adipocyte associated 1	−1.097353862	7.10679E-39
ENSG00000138111	MFSD13A	Major facilitator superfamily domain containing 13A	−1.081598123	7.26073E-13
ENSG00000198794	SCAMP5	Secretory carrier membrane protein 5	−1.069454475	1.43271E-14
ENSG00000141543	EIF4A3	Eukaryotic translation initiation factor 4A3	−1.053239423	5.13661E-21
ENSG00000102230	PCYT1B	Phosphate cytidylyltransferase 1B, choline	−1.046421228	4.22045E-08
ENSG00000135097	MSI1	Musashi RNA binding protein 1	−1.037804441	5.79642E-10
ENSG00000183624	HMCES	5-hydroxymethylcytosine binding, ES cell specific	−1.03418709	5.91112E-12
ENSG00000144043	TEX261	Testis expressed 261	−1.018612109	1.10457E-43
ENSG00000094804	CDC6	Cell division cycle 6	−1.014134633	1.72692E-25
ENSG00000197147	LRRC8B	Leucine rich repeat containing 8 VRAC subunit B	−0.998151811	4.25272E-13
ENSG00000102977	ACD	ACD shelterin complex subunit and telomerase recruitment factor	−0.990099933	6.25433E-18
ENSG00000275591	XKR5	XK related 5	−0.988185209	0.002695943
ENSG00000176454	LPCAT4	Lysophosphatidylcholine acyltransferase 4	−0.985862564	2.7143E-15
ENSG00000173653	RCE1	Ras converting CAAX endopeptidase 1	−0.984715649	7.9172E-26
ENSG00000007062	PROM1	Prominin 1	−0.97961611	0.009103299
ENSG00000104369	JPH1	Junctophilin 1	−0.977497844	8.80282E-09
ENSG00000180998	GPR137C	G protein-coupled receptor 137C	−0.974003522	0.00222764
ENSG00000178999	AURKB	Aurora kinase B	−0.966160873	4.06418E-33
ENSG00000103502	CDIPT	CDP-diacylglycerol--inositol 3-phosphatidyltransferase	−0.951329558	3.04278E-22
ENSG00000196081	ZNF724	Zinc finger protein 724	−0.950308152	0.013396392
ENSG00000127220	ABHD8	Abhydrolase domain containing 8	−0.944454528	6.54254E-10
ENSG00000162517	PEF1	Penta-EF-hand domain containing 1	−0.939626338	1.33625E-15
ENSG00000172731	LRRC20	Leucine rich repeat containing 20	−0.935106976	1.72682E-11
ENSG00000088881	EBF4	EBF family member 4	−0.933391831	0.034477292
ENSG00000167700	MFSD3	Major facilitator superfamily domain containing 3	−0.928218276	1.883E-16
ENSG00000109079	TNFAIP1	TNF alpha induced protein 1	−0.916384936	4.07361E-23
ENSG00000183726	TMEM50A	Transmembrane protein 50A	−0.909466682	2.40125E-14
ENSG00000093009	CDC45	Cell division cycle 45	−0.907262883	4.28719E-19
ENSG00000233927	RPS28	Ribosomal protein S28	−0.897534175	8.88364E-14
ENSG00000007968	E2F2	E2F transcription factor 2	−0.89724828	2.11235E-14
ENSG00000163964	PIGX	Phosphatidylinositol glycan anchor biosynthesis class X	−0.893049786	6.33205E-11
ENSG00000197696	NMB	Neuromedin B	−0.881664148	0.000489433
ENSG00000160767	FAM189B	Family with sequence similarity 189 member B	−0.878099585	3.43974E-32
ENSG00000128408	RIBC2	RIB43A domain with coiled-coils 2	−0.876289923	2.7777E-05
ENSG00000131873	CHSY1	Chondroitin sulfate synthase 1	−0.876260038	3.45611E-27
ENSG00000130830	MPP1	Membrane palmitoylated protein 1	−0.863737396	1.43029E-11
ENSG00000128604	IRF5	Interferon regulatory factor 5	−0.859313035	0.027699243
ENSG00000065328	MCM10	Minichromosome maintenance 10 replication initiation factor	−0.85800114	8.95999E-16
ENSG00000100139	MICALL1	MICAL like 1	−0.850088943	3.82816E-22
ENSG00000146094	DOK3	Docking protein 3	−0.849727315	0.01351518
ENSG00000099617	EFNA2	Ephrin A2	−0.84965942	0.000183769
ENSG00000008118	CAMK1G	Calcium/calmodulin dependent protein kinase IG	−0.839801505	8.3045E-06
ENSG00000109084	TMEM97	Transmembrane protein 97	−0.835177517	3.98655E-15
ENSG00000104524	PYCR3	Pyrroline-5-carboxylate reductase 3	−0.833707143	3.48264E-17
ENSG00000181991	MRPS11	Mitochondrial ribosomal protein S11	−0.828776036	1.70901E-20
ENSG00000197312	DDI2	DNA damage inducible 1 homolog 2	−0.821175254	1.12384E-11
ENSG00000131504	DIAPH1	Diaphanous related formin 1	−0.818668953	3.61781E-30
ENSG00000141401	IMPA2	Inositol monophosphatase 2	−0.8179472	3.21857E-09
ENSG00000007541	PIGQ	Phosphatidylinositol glycan anchor biosynthesis class Q	−0.815324807	3.82816E-22
ENSG00000039068	CDH1	Cadherin 1	−0.813795041	0.007133781
ENSG00000136052	SLC41A2	Solute carrier family 41 member 2	−0.809068678	0.000110523
ENSG00000006625	GGCT	Gamma-glutamylcyclotransferase	−0.808252948	7.97127E-08
ENSG00000159259	CHAF1B	Chromatin assembly factor 1 subunit B	−0.803311835	4.58958E-17
ENSG00000135622	SEMA4F	Semaphorin 4F	−0.79905126	3.1063E-11
ENSG00000003989	SLC7A2	Solute carrier family 7 member 2	−0.798981174	1.70805E-06
ENSG00000111199	TRPV4	Transient receptor potential cation channel subfamily V member 4	−0.795894675	0.026723176
ENSG00000111666	CHPT1	Choline phosphotransferase 1	−0.790075556	8.37199E-07
ENSG00000132563	REEP2	Receptor accessory protein 2	−0.789760272	0.017578955
ENSG00000166803	PCLAF	PCNA clamp associated factor	−0.787731791	2.9663E-10
ENSG00000103018	CYB5B	Cytochrome b5 type B	−0.782156755	6.96841E-07
ENSG00000205309	NT5M	5′,3′-nucleotidase, mitochondrial	−0.779227105	0.015346667
ENSG00000163888	CAMK2N2	Calcium/calmodulin dependent protein kinase II inhibitor 2	−0.777929574	0.004548607
ENSG00000167272	POP5	POP5 homolog, ribonuclease P/MRP subunit	−0.776677827	8.58558E-11
ENSG00000112039	FANCE	FA complementation group E	−0.776465629	1.15064E-10
ENSG00000165046	LETM2	Leucine zipper and EF-hand containing transmembrane protein 2	−0.775536248	0.00139097
ENSG00000254087	LYN	LYN proto-oncogene, Src family tyrosine kinase	−0.775426645	2.03074E-15
ENSG00000267534	S1PR2	Sphingosine-1-phosphate receptor 2	−0.773810704	4.2899E-09
ENSG00000146670	CDCA5	Cell division cycle associated 5	−0.773646868	3.06923E-17
ENSG00000137404	NRM	Nurim	−0.772726067	8.13588E-20
ENSG00000116791	CRYZ	Crystallin zeta	−0.772545614	0.000174379
ENSG00000102384	CENPI	Centromere protein I	−0.769337411	2.02709E-10
ENSG00000152270	PDE3B	Phosphodiesterase 3B	−0.764313087	4.05836E-07
ENSG00000089063	TMEM230	Transmembrane protein 230	−0.760695783	4.69492E-08
ENSG00000187123	LYPD6	LY6/PLAUR domain containing 6	−0.760114435	3.36599E-09
ENSG00000117408	IPO13	Importin 13	−0.759202881	3.22313E-19
ENSG00000124587	PEX6	Peroxisomal biogenesis factor 6	−0.758743154	2.95953E-09
ENSG00000140365	COMMD4	COMM domain containing 4	−0.752311848	9.66051E-19
ENSG00000144935	TRPC1	Transient receptor potential cation channel subfamily C member 1	−0.749164557	0.011475086
ENSG00000125319	HROB	Homologous recombination factor with OB-fold	−0.747598102	7.23784E-12
ENSG00000170515	PA2G4	Proliferation-associated 2G4	−0.747546163	8.61016E-16
ENSG00000107796	ACTA2	Actin alpha 2, smooth muscle	−0.742851497	1.12389E-05
ENSG00000075218	GTSE1	G2 and S-phase expressed 1	−0.741904138	8.50653E-14
ENSG00000169105	CHST14	Carbohydrate sulfotransferase 14	−0.740579126	6.41331E-17
ENSG00000276043	UHRF1	Ubiquitin like with PHD and ring finger domains 1	−0.736980602	4.32998E-10
ENSG00000167005	NUDT21	Nudix hydrolase 21	−0.735118324	6.40005E-08
ENSG00000165806	CASP7	Caspase 7	−0.733408293	1.08616E-07
ENSG00000159147	DONSON	DNA replication fork stabilization factor DONSON	−0.733330798	1.79372E-08
ENSG00000137310	TCF19	Transcription factor 19	−0.73287457	2.6109E-11
ENSG00000188186	LAMTOR4	Late endosomal/lysosomal adaptor, MAPK and MTOR activator 4	−0.731023031	3.67345E-05
ENSG00000189057	FAM111B	FAM111 trypsin like peptidase B	−0.729814217	3.26705E-06
ENSG00000113119	TMCO6	Transmembrane and coiled-coil domains 6	−0.729753081	3.77511E-08
ENSG00000132016	BRME1	Break repair meiotic recombinase recruitment factor 1	−0.729700409	0.001873868
ENSG00000160753	RUSC1	RUN and SH3 domain containing 1	−0.728484775	5.50404E-17
ENSG00000166508	MCM7	Minichromosome maintenance complex component 7	−0.728365624	2.96948E-16
ENSG00000221955	SLC12A8	Solute carrier family 12 member 8	−0.726823055	0.039726441
ENSG00000109519	GRPEL1	GrpE like 1, mitochondrial	−0.725689313	6.80513E-09
ENSG00000123685	BATF3	Basic leucine zipper ATF-like transcription factor 3	−0.72458263	0.001522053
ENSG00000148110	MFSD14B	Major facilitator superfamily domain containing 14B	−0.722394583	1.8746E-13
ENSG00000177917	ARL6IP6	ADP ribosylation factor like GTPase 6 interacting protein 6	−0.718700533	1.42364E-08
ENSG00000180228	PRKRA	Protein activator of interferon induced protein kinase EIF2AK2	−0.716677371	2.02939E-12
ENSG00000136574	GATA4	GATA binding protein 4	−0.711352562	0.007185606
ENSG00000077152	UBE2T	Ubiquitin conjugating enzyme E2 T	−0.709554446	1.08386E-14
ENSG00000183010	PYCR1	Pyrroline-5-carboxylate reductase 1	−0.708477376	3.11792E-10
ENSG00000146648	EGFR	Epidermal growth factor receptor	−0.70834571	0.001003247
ENSG00000085840	ORC1	Origin recognition complex subunit 1	−0.707703656	1.16587E-11
ENSG00000064393	HIPK2	Homeodomain interacting protein kinase 2	−0.706969488	1.76206E-14
ENSG00000141542	RAB40B	RAB40B, member RAS oncogene family	−0.703840844	0.001296392
ENSG00000143590	EFNA3	Ephrin A3	−0.703826254	0.004199258
ENSG00000105676	ARMC6	Armadillo repeat containing 6	−0.702557762	2.88567E-20
ENSG00000130734	ATG4D	Autophagy related 4D cysteine peptidase	−0.700560962	7.30284E-14
ENSG00000176974	SHMT1	Serine hydroxymethyltransferase 1	−0.700145922	2.87823E-09
ENSG00000168792	ABHD15	Abhydrolase domain containing 15	−0.699505212	7.30284E-14
ENSG00000163577	EIF5A2	Eukaryotic translation initiation factor 5A2	−0.698832403	5.90333E-07
ENSG00000149636	DSN1	DSN1 component of MIS12 kinetochore complex	−0.69800473	1.72175E-09
ENSG00000054277	OPN3	Opsin 3	−0.697429021	0.005367454
ENSG00000158109	TPRG1L	Tumor protein p63 regulated 1 like	−0.69576735	7.88947E-12
ENSG00000007255	TRAPPC6A	Trafficking protein particle complex subunit 6A	−0.69333341	0.003909706
ENSG00000186318	BACE1	Beta-secretase 1	−0.693003562	1.59805E-10
ENSG00000123444	KBTBD4	Kelch repeat and BTB domain containing 4	−0.691858123	1.14532E-08
ENSG00000129173	E2F8	E2F transcription factor 8	−0.691368268	1.53483E-07
ENSG00000116337	AMPD2	Adenosine monophosphate deaminase 2	−0.688901876	7.65844E-20
ENSG00000034152	MAP2K3	Mitogen-activated protein kinase kinase 3	−0.688418607	7.14337E-12
ENSG00000188312	CENPP	Centromere protein P	−0.687348522	1.41132E-07
ENSG00000101412	E2F1	E2F transcription factor 1	−0.685900049	1.57498E-11
ENSG00000178752	ERFE	Erythroferrone	−0.685630344	8.2682E-07
ENSG00000167513	CDT1	Chromatin licensing and DNA replication factor 1	−0.684859075	4.78247E-12
ENSG00000076248	UNG	Uracil DNA glycosylase	−0.684741771	9.43088E-09
ENSG00000143416	SELENBP1	Selenium binding protein 1	−0.684686569	3.17263E-08
ENSG00000144554	FANCD2	FA complementation group D2	−0.684496681	2.65731E-14
ENSG00000167524	RSKR	Ribosomal protein S6 kinase related	−0.684436037	0.040929878
ENSG00000170085	SIMC1	SUMO interacting motifs containing 1	−0.684107509	4.2041E-08
ENSG00000160233	LRRC3	Leucine rich repeat containing 3	−0.681849404	1.74181E-07
ENSG00000157927	RADIL	Rap associating with DIL domain	−0.67922331	2.07827E-05
ENSG00000136463	TACO1	Translational activator of cytochrome c oxidase I	−0.678764049	0.000179835
ENSG00000136146	MED4	Mediator complex subunit 4	−0.677778412	2.74481E-12
ENSG00000274641	H2BC17	H2B clustered histone 17	−0.676395464	1.28072E-07
ENSG00000140534	TICRR	TOPBP1 interacting checkpoint and replication regulator	−0.675488889	3.02285E-10
ENSG00000076003	MCM6	Minichromosome maintenance complex component 6	−0.675329486	1.48125E-10
ENSG00000187741	FANCA	FA complementation group A	−0.672648133	1.15152E-09
ENSG00000276368	H2AC14	H2A clustered histone 14	−0.672072742	2.72213E-06
ENSG00000106236	NPTX2	Neuronal pentraxin 2	−0.671365	0.034171406
ENSG00000111331	OAS3	2′-5′-oligoadenylate synthetase 3	−0.669574335	0.000154628
ENSG00000180011	ZADH2	Zinc binding alcohol dehydrogenase domain containing 2	−0.66559808	9.40295E-07
ENSG00000134987	WDR36	WD repeat domain 36	−0.664728619	3.40613E-09
ENSG00000116685	KIAA2013	KIAA2013	−0.66440737	1.57824E-10
ENSG00000131153	GINS2	GINS complex subunit 2	−0.663138222	3.08693E-10
ENSG00000165480	SKA3	Spindle and kinetochore associated complex subunit 3	−0.661381525	1.68765E-09
ENSG00000070366	SMG6	SMG6 nonsense mediated mRNA decay factor	−0.660870628	2.32961E-09
ENSG00000100401	RANGAP1	Ran GTPase activating protein 1	−0.660517742	6.83745E-16
ENSG00000169884	WNT10B	Wnt family member 10B	−0.657739715	7.54512E-06
ENSG00000004864	SLC25A13	Solute carrier family 25 member 13	−0.657514506	5.50404E-17
ENSG00000179532	DNHD1	Dynein heavy chain domain 1	−0.657360294	0.000903431
ENSG00000169679	BUB1	BUB1 mitotic checkpoint serine/threonine kinase	−0.65732404	1.12205E-15
ENSG00000092853	CLSPN	Claspin	−0.657027473	8.82631E-11
ENSG00000175175	PPM1E	Protein phosphatase, Mg2 + /Mn2+ dependent 1E	−0.656944979	0.009390568
ENSG00000128973	CLN6	CLN6 transmembrane ER protein	−0.656174514	1.45612E-13
ENSG00000132646	PCNA	Proliferating cell nuclear antigen	−0.654414489	2.43078E-10
ENSG00000136982	DSCC1	DNA replication and sister chromatid cohesion 1	−0.653987621	3.70426E-09
ENSG00000102387	TAF7L	TATA-box binding protein associated factor 7 like	−0.652644895	0.028474932
ENSG00000160957	RECQL4	RecQ like helicase 4	−0.652136684	2.0202E-12
ENSG00000160117	ANKLE1	Ankyrin repeat and LEM domain containing 1	−0.651964708	0.012275559
ENSG00000127564	PKMYT1	Protein kinase, membrane associated tyrosine/threonine 1	−0.650369204	1.59377E-11
ENSG00000116199	FAM20B	FAM20B glycosaminoglycan xylosylkinase	−0.650171171	2.25423E-05
ENSG00000148985	PGAP2	Post-GPI attachment to proteins 2	−0.650053227	4.6646E-10

### SETDB1 inhibition triggers DNA damage and senescence-like phenotypes

DNA replication takes place in the S phase of the cell cycle. To further analyze the S phase entry, OMM1.3 proliferating cells treated with control siRNA or siRNA against SETDB1 were stained for incorporated EdU against total DNA content using Hoechst 33342. SETDB1-KD confirmed by immunoblot (Supplementary Fig. 3A), displayed increased percentage of cells in G0/G1 phase and reduced percentage of cells in late S phase upon flow cytometry analyses and quantification (Supplementary Fig. 3B–D).

Given that accurate and complete DNA replication is critical for achieving genome integrity and cell survival, and that eukaryotic cells progressing through S phase with a reduced number of licensed origins are more vulnerable to replication stress and DNA damage, we hypothesized that SETDB1 downregulation would promote DNA damage. To address this point, we analyzed phosphorylated CHK2 and H2AX (γ-H2AX), canonical markers of DNA damage and checkpoint activation. We also performed staining of 53BP1, a marker of DNA double-strand breaks and an important component of the DNA damage response [19].

**Fig. 3:**
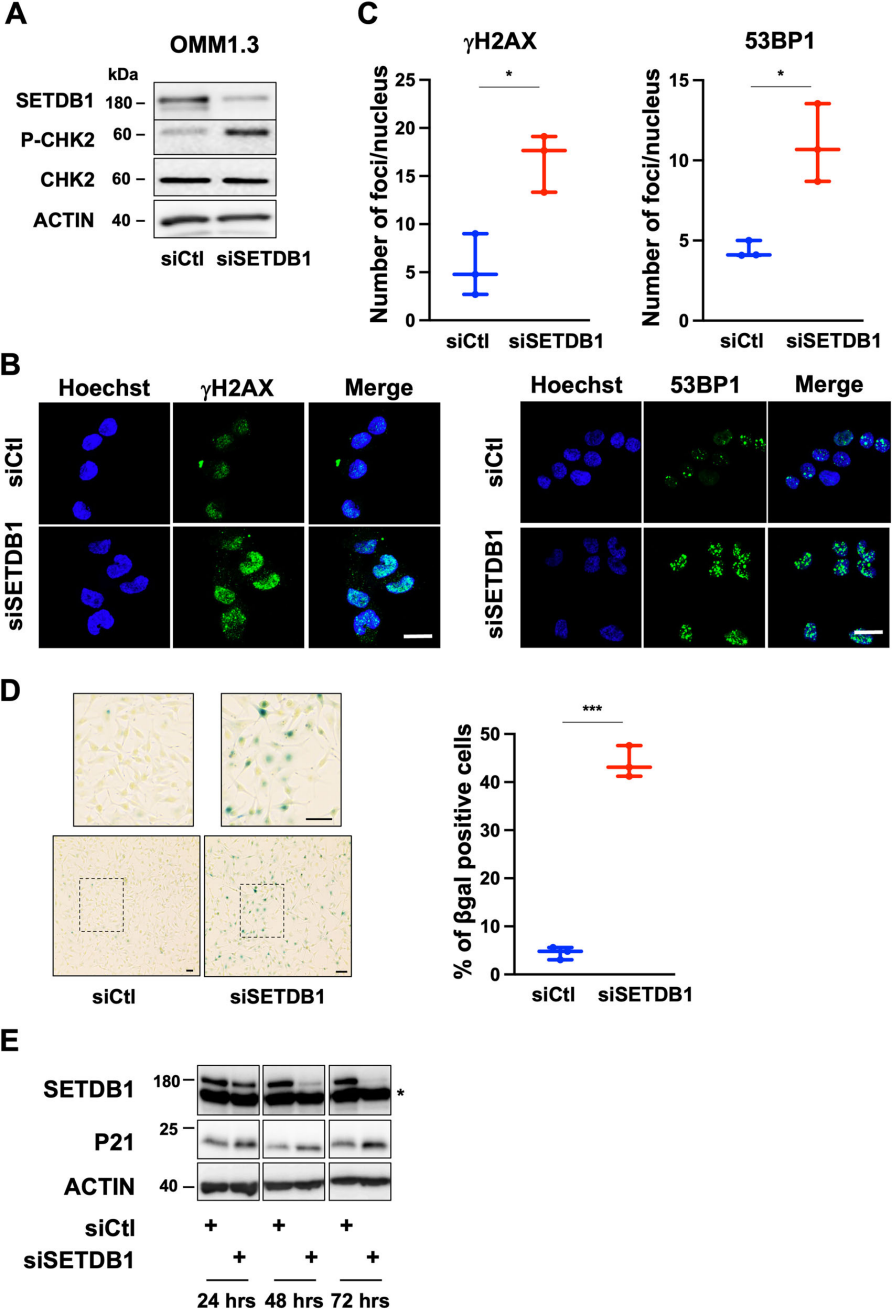
SETDB1 knockdown triggers DNA damage and senescence-like phenotypes. **A** Immunoblot to SETDB1, CHK2, and P-CHK2 in lysates of OMM1.3 cells treated with control siRNA or siRNA to SETDB1 for 48 h. β-Actin is used as a loading control. **B** OMM1.3 cells treated with control siRNA or siRNA to SETDB1 for 72 h were analyzed by immunofluorescence for H2AX phosphorylated on Ser139 (γH2AX) or 53BP1. Cell nuclei were counterstained with Hoechst. Representative fluorescence images are shown. Bar = 20 µM. **C** Representative box and whiskers plots of quantification of γH2AX (left, *n* = 3. *p*-value was derived from Welch’s *t*-test. *p = 0.012) or 53BP1 (right, *n* = 3. *p*-value was derived from Welch’s *t*-test. *p = 0.038) foci number per nucleus. **D** Senescence-associated β-galactosidase (SA-β-Gal) staining of OMM1.3 cells treated with control siRNA or siRNA to SETDB1 for 96 h, Bar = 20 µM (left) and representative box and whiskers plots of quantification with the percentage of SA-β-Gal positive cells relative to the total number of cells (right) *n* = 3. *p*-value was derived from Welch’s *t*-test. ***p = 0.0007. **E** Immunoblot to SETDB1 and p21 of OMM1.3 cells treated with control siRNA or siRNA to SETDB1 for 24, 48, and 72 h. β-Actin was used as a loading control. * Indicates non-specific band.

Immunoblot of OMM1.3 cells treated with siRNA to SETDB1 showed enhanced phosphorylation of CHK2 (Fig. 3A). Moreover, as shown by immunofluorescence analyses, SETDB1-KD enhanced γ-H2AX and 53BP1 foci formation compared to the control cells (Fig. 3B, C). Enhanced phosphorylation of CHK2 as well as an increased γ-H2AX and 53BP1 staining, were also observed when expression of SETDB1 was reduced using the CRISPR-Cas9 approach as compared to cells infected with control sgRNA (Supplementary Fig. 4A–D). Together, these data indicate that SETDB1 KD is sufficient to drive the DNA damage response. Persistent DNA damage is well known to promote a senescent phenotype [20]. Thus, we then conducted β-Galactosidase (SA-βGal) staining at pH6 to measure senescence. Our data demonstrated SA-βGal staining in OMM1.3 treated with SETDB1 siRNA compared to control cells (Fig. 3D) as well as using a sgRNA against SETDB1 (Supplementary Fig. 4E). SA-βGal staining following SETDB1 KD by siRNA was confirmed in OMM1 cells (Supplementary Fig. 5A, B). In addition, time course analysis showed that SETDB1 KD caused enhanced p21 expression another marker of the senescence state and of growth arrest (Fig. 3E) that is observed later on. Collectively, our findings indicate that SETDB1 expression may sustain DNA replication and prevent DNA damage to overcome the process of senescence and favor uveal melanoma cell growth.

### CDC6 depletion phenocopies SETDB1 knockdown

**Fig. 4:**
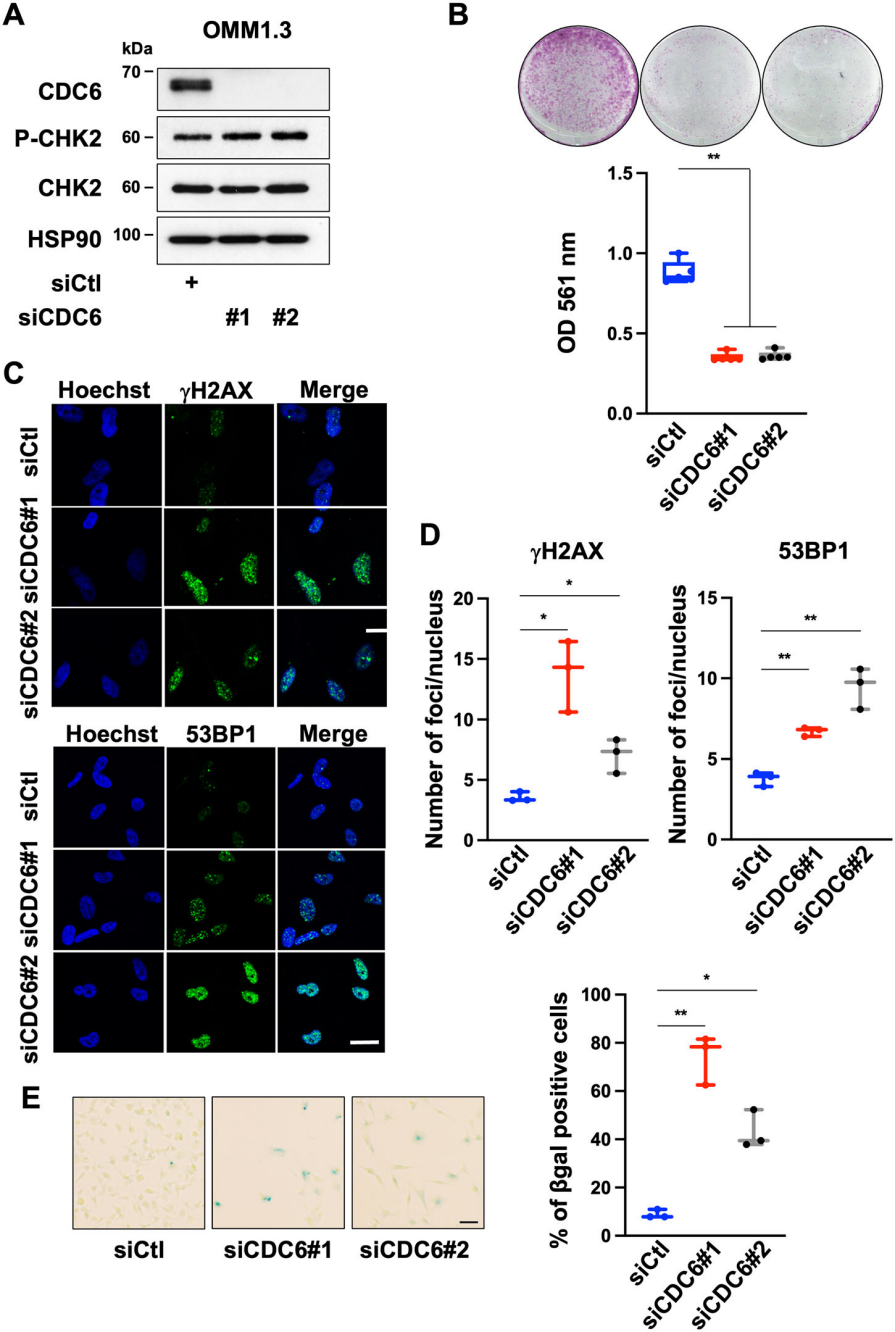
CDC6 knockdown induces growth arrest and senescence-like features. **A** Immunoblot to CDC6, CHK2, and P-CHK2 in lysates of OMM1.3 cells treated with a control siRNA or with two different siRNA to CDC6 for 72 h. HSP90 was used as a loading control. **B** OMM1.3 cells treated with a control siRNA (siCtl) or siRNA to CDC6 were seeded at the same density and cultured for 10 days (top), representative box and whiskers plots of crystal violet quantification at OD 561 nm (bottom). Mann–Whitney test was performed for comparison between groups, *n* = 5. Data are mean ± SEM. **p = 0.0079. **C** OMM1.3 cells treated for 72 h with control siRNA or siRNA to CDC6 were analyzed by immunofluorescence for H2AX phosphorylated on Ser139 (γH2AX) or 53BP1. Cell nuclei were counterstained with Hoechst. Representative fluorescence images are shown. Bar = 20 µM. **D** Representative box and whiskers plots of quantification of γH2AX (left, *n* = 3. *p*-value was derived from Welch’s *t*-test. *p = 0.025, *p = 0.041) or 53BP1 (right, *n* = 3. *p*-value was derived from Welch’s *t*-test. **p = 0.013, **p = 0.009) foci number per nucleus. **E** SA-β-Gal staining of OMM1.3 cells treated with control siRNA or siRNA to CDC6 for 6 days. Bar=20 µM (left) and representative box and whiskers plots of quantification with the percentage of SA-β-Gal positive cells relative to the total number of cells (right) *n* = 3. *p*-value was derived from Welch’s *t*-test. **p = 0.0068, *p = 0.0139.

Cell division cycle 6 (*CDC6*) and Minichromosome Maintenance Complex Component 6 (MCM6), that are downregulated in cells with reduced SETDB1 level, are essential regulators of DNA replication in eukaryotic cells and they play critical roles in the establishment and maintenance of the cell replication program [21]. However, OMM1.3 and OMM2.5 cells treated with siRNA against MCM6 did not display reduced growth potential (Supplementary Fig. 6A–D). In contrast, CDC6 depletion with two different siRNAs reduced cell growth capacity of OMM1.3 cells (Fig. 4A, B). This finding was extended to OMM2.5 cells treated with the CDC6 siRNAs, which also exhibited reduced cell growth capacity (Supplementary Fig. 7A, B). These data prompted us to focus our attention on CDC6. As performed for SETDB1, we analyzed phosphorylated CHK2, H2AX (γ-H2AX) and 53BP1. Immunoblot of OMM1.3 cells treated with siRNAs to CDC6 showed enhanced phosphorylation of CHK2 (Fig. 4A) and immunofluorescence revealed that CDC6 KD activated the DNA damage response as illustrated by enhanced γ-H2AX and 53BP1 staining compared to control cells (Fig. 4C, D). SA-βGal activity was also detected in cells treated with CDC6 siRNAs (Fig. 4E). Altogether, our findings demonstrate the critical role of CDC6 in DNA replication, but also in DNA repair and senescence and suggest that CDC6 function downstream SETDB1 in uveal melanoma cell growth.

### Anti-SETDB1 therapy reduces uveal melanoma cell growth

**Fig. 5:**
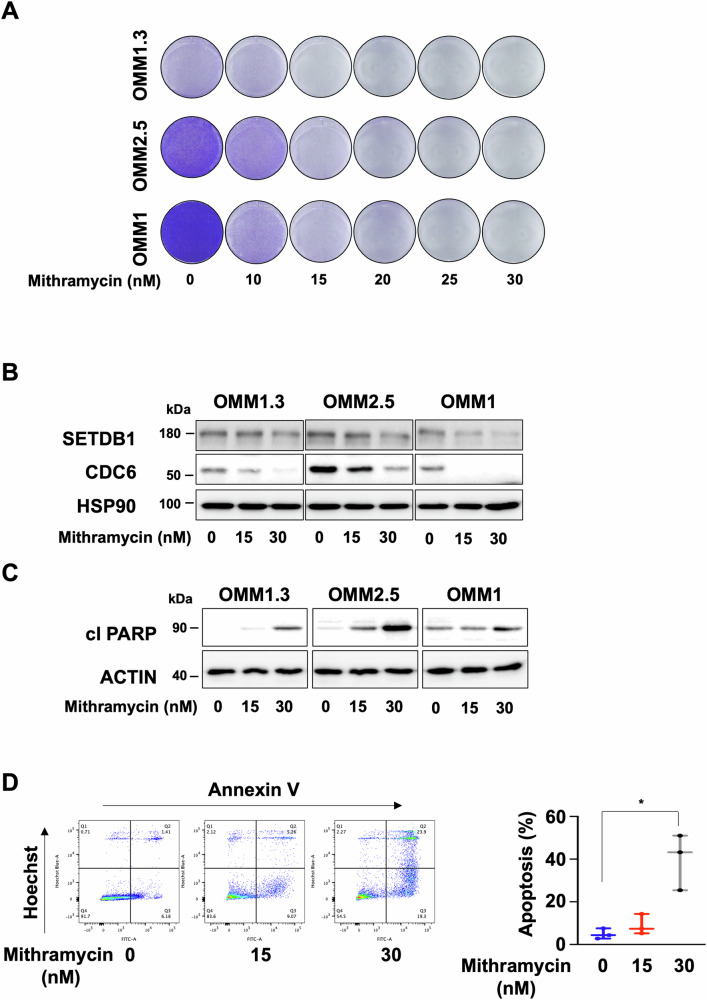
Anti-SETDB1 therapy reduces metastatic uveal melanoma cell proliferation and survival. **A** OMM1.3, OMM2.5, and OMM1 metastatic uveal melanoma cells were seeded at low density and cultured for 96 h in absence or presence of increasing concentration of Mithramycin A. Representative images of three independent experiments are shown. **B** Immunoblot analysis of metastatic uveal melanoma cells exposed to Mithramycin A (15 and 30 nM) for 72 h with the indicated antibodies. HSP90 was used as a loading control. **C** Immunoblot to cleaved PARP in control OMM1.3 cells and OMM1.3 cells treated with Mithramycin A (15 and 30 nM) for 72 h. β-Actin was used as a loading control. **D** Analysis of apoptosis in control OMM1.3 cells and OMM1.3 cells treated with Mithramycin A at the indicated concentrations for 96 h. Annexin V diagram and quantification of the percentage of late apoptotic cells using the Annexin V assay, *n* = 3. *p*-value was derived from Welch’s *t*-test. *p = 0.0399.

Our data point towards SETDB1 as a potential relevant therapeutic target in metastatic uveal melanomas. Mithramycin A, an antitumor antibiotic used in phase II clinical trials for the treatment of patients with a broad range of malignancies (ClinicalTrials.gov: NCT01624090), has been demonstrated to inhibit SETDB1 expression [22]. Mithramycin A is reported to impair SETDB1 expression by blocking binding of the SP-1 transcription factor at the *SETDB1* promoter [23]. We tested the effect of Mithramycin A on different GNAQ/11-mutated human metastatic uveal melanoma cells in vitro. OMM1.3 cells exposed to increasing concentration of Mithramycin A showed reduced cell growth ability (Fig. 5A). Similar observations were performed in two other human metastatic uveal cell lines, OMM2.5 and OMM1, demonstrating that the growth defects were not restrain to a unique cell line (Fig. 5A). Interestingly, a more direct SETDB1 inhibitor (SETDB1i), which prevents the interaction of SETDB1 with histones, has been recently reported [24]. Increasing concentration of SETDB1i also caused reduced cell growth ability of the different human metastatic uveal melanoma cell lines (Supplementary Fig. 8A). Therefore, our data show that a panel of human metastatic uveal melanoma cells are sensitive to two different SETDB1 inhibitors.

Both Mithramycin A and SETDB1i reduced SETDB1 and CDC6 protein levels (Fig. 5B and supplementary Fig. 8B), in agreement with CDC6 being regulated by SETDB1. It is worth noting that Mithramycin A and SETDB1i did not induce SA-βGal staining. Instead, they both promoted apoptosis as illustrated by annexin V/PI labelling and the detection of cleaved PARP, a well-studied caspase 3 substrate. It is also worth noting that apoptosis was most visible at the highest inhibitor concentration (Fig. 5C, D and Supplementary Fig. 8C, D).

**Fig. 6:**
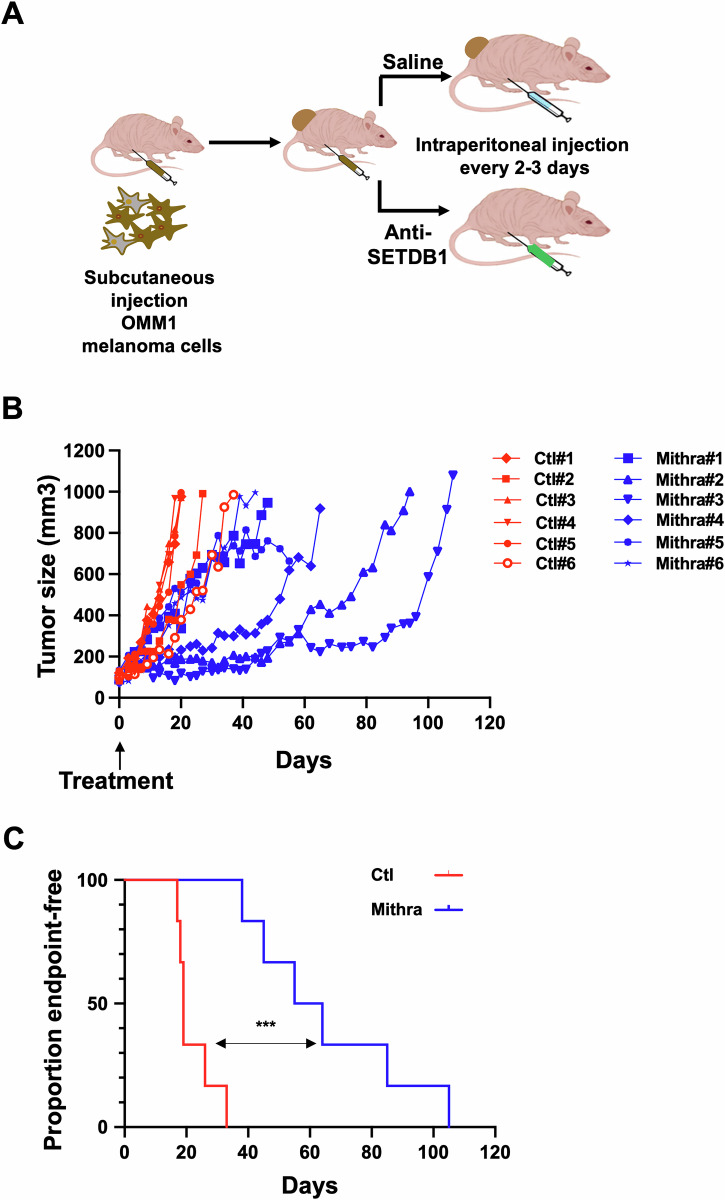
SETDB1 inhibition has anti-tumoral effect in vivo. **A** Schematic of in vivo experiment where NSG mice were injected subcutaneously with OMM1 cells. When the tumor reached 100 mm^3^, mice began a treatment regimen of vehicle (PBS) or Mithramycin A via intraperitoneal injection (1 mg/kg) three times per week. **B** Plot showing tumor volume for individual mice bearing a OMM1 cell xenograft treated with PBS (Control mice in red) or with Mithramycin A (Mithramycin A-treated mice in blue). **C** Kaplan–Meier analyses of tumor growth to ethical endpoint for mice bearing the OMM1 cell xenografts showing time to 0.8 cm^3^ in vehicle (red) or 1 mg/kg Mithramycin A (blue) treated mice. Log-rank (Mantel–Cox) test was performed for comparison between groups. ***p = 0.0005.

These data indicate that SETDB1 represents a promising anti-metastatic uveal melanoma therapy. To address this point, we next investigated the therapeutic relevance of inhibiting SETDB1 on tumor growth in vivo. Given that Mithramycin A works at nanomolar concentration compared to SETDB1i and that it has been assessed in clinical trials, we pursued in vivo studies with Mithramycin A. Notably, no toxicity was observed in normal uveal melanocytes or fibroblasts (Supplementary Fig. 9A, B). Both male and female mice were used in these experiments since uveal melanoma incidence is similar in men and women. OMM1 cells were subcutaneously injected into NSG immunodeficient mice and when the tumors were palpable (approximately 100 mm^3^), mice were injected intraperitoneally every 3 days with Mithramycin A (1 mg/kg) (Fig. 6A). When the tumor reached *~*1 cm^3^, the mouse was sacrificed. Our data show that Mithramycin A strongly impaired uveal melanoma growth as illustrated by reduced tumor volume compared to the vehicle control group (Fig. 6B). Moreover, treatment with Mithramycin A resulted in a significant survival advantage until the ethical endpoint was reached compared to vehicle-treated mice (Fig. 6C) and no metastases were observed in this experiment.

Altogether, our findings demonstrate that SETDB1 critically supports metastatic uveal melanoma progression in vivo, and establish SETDB1 as a promising effective therapeutic strategy in these often untreatable tumors.

## Discussion

We hereby present a chromatin-focused CRISPR-Cas9 screen to identify factors that play a critical role in proliferation and survival of metastatic uveal melanoma cells, and identified the lysine methyltransferase SETDB1. Our screen also revealed additional candidates. In our context, the histone demethylase KDM5C that removes the active H3K4me3 marks and the histone methyltransferase PRMT6 adding the repressive H3R2me2a marks could contribute to tumor suppressor gene silencing. The histone demethylase KDM4D targeting the repressive H3K9me2/H3K9me3 marks and the histone methyltransferase SETD1B, which deposits activating H3K4me3 marks might activate oncogenes [25, 26]. These regulators might create aberrant chromatin landscape in uveal melanoma cells in which their roles remain to be elucidated, thus representing a new area for future investigation.

SETDB1 has been reported to be upregulated in a variety of tumors, such as human skin melanomas, and promotes cancer development [27]. Previous work also showed that SETDB1 regulates the proliferation of different tumor cells in vitro and in vivo [28–32]. Aligned with that, our findings show a high expression of SETDB1 protein in human uveal melanoma cells compared to normal uveal melanocytes. We also found that SETDB1 knockdown in uveal melanomas activates a DNA damage response, associated with a senescence-like state and a growth arrest. This senescence-like state has been shown to be overcame by SETDB1 overexpression in the study of Ceol and collaborators, most likely through a different mechanism that we observed [27]. Indeed, among the list of genes downregulated in *SETDB1* overexpressing melanomas that they have identified, none are deregulated in our transcriptomic data. These data indicate that SETDB1 plays a critical role in growth of uveal melanoma cells, likely by overcoming the process of senescence, a major barrier against tumor progression, most likely in a context-dependent function.

SETDB1 is amplified in different types of cancers, including breast, ovarian, bladder, and cutaneous melanomas, in which it can also be found mutated [27, 31, 33]. We found a higher SETDB1 level in metastatic uveal melanoma cells compared to normal choroidal melanocytes, yet analysis of public datasets revealed no alteration in copy number or mutations in SETDB1 in uveal melanomas. However, increased SETDB1 expression could occur through other mechanisms, including chromosomal translocation, single-nucleotide polymorphism in regulatory regions and mutation or activation of upstream signaling pathways. How SETDB1 expression is regulated in uveal melanoma remains to be determined.

A metastasis-promoting role for SETDB1 has also been reported in different cancer types, such as in colorectal cancer, and in cutaneous melanomas [29, 34] in which high SETDB1 expression was detected at the invasive front [28]. Supporting this, in cutaneous malignant melanomas, SETDB1 regulates the expression of thrombospondin-1, known to stimulate metastasis formation [28]. Our data did not show any change in the motile ability of SETDB1 knocked-down uveal melanoma cells (not shown). In contrast, our transcriptomic analysis highlighted downregulation of factors involved in the assembly of the replication initiation machinery in SETDB1-deficient cells. Among them, reduced CDC6 and MCM6 expression was validated at the protein level, but only CDC6 knockdown impaired metastatic uveal melanoma cell growth. The effect of MCM6 reduction is likely to be offset by the high abundance of MCM proteins in contrast to CDC6, which is an essential and rate-limiting factor [35, 36]. High CDC6 expression is associated with enhanced malignant behavior of cancer cells [37, 38] and drug resistance [39–41]. Upon CDC6 inhibition, uveal melanoma cells exhibited signs of DNA damage and senescent phenotypes. Growth inhibition and senescence in response to CDC6 inhibition have been evoked in other cancers [42, 43]. Altogether, our observations suggest that SETDB1 can mediate its effect in part through CDC6.

Hence, both SETDB1 and CDC6 might represent new prognostic biomarkers and new potential therapeutic targets in uveal melanomas.

How SETDB1 inhibits the expression of these factors remains to be elucidated. It is known to catalyze the repressive H3K9me3 mark and mediate gene repression. However, we observed no difference in overall H3K9me2/3 levels after SETDB1 knockdown in OMM1.3 uveal melanoma cells. We cannot rule out the existence of compensation mechanisms by other H3K9 methyltransferases for the regulation of overall H3K9me3 metabolism in metastatic uveal melanoma cells. Indeed, H3K9me3 is catalyzed by two enzymatic systems, SUV39H and SETDB1/ESET1 (SET domain bifurcated) [44]. Another possibility is that SETDB1 regulates a transcriptional program independently of its H3K9me3 activity. This is reminiscent of other studies demonstrating that SETDB1 functions through methylation of non-histone proteins such as p53 or AKT [45, 46].

Regardless of the precise mechanisms of SETDB1 activity, given that SETDB1 inhibition strongly reduced the growth capacity of metastatic uveal melanoma cells, suggests that SETDB1 is a highly relevant therapeutic target for the treatment of this tumor type.

This is evidenced by in vitro effect of Mithramycin A and SETDB1i, two reported SETDB1 inhibitors. It is noteworthy that Mithramycin A or SETDB1i does not induce senescence but instead apoptosis. This might be related to the levels of p53-p21 pathway activation. Importantly, in a pre-clinical model, Mithramycin A strongly reduced metastatic uveal melanoma progression and robustly extended mouse lifespan.

In sum, our findings demonstrate that SETDB1 inhibition represent a novel and valid therapeutic option for the treatment of metastatic uveal melanomas.

## Material and Methods

### Cell cultures

Human uveal melanoma cell lines OMM1.3 (GNAQ^Q209P^) [47] and OMM2.5 (GNAQ^Q209P^) [47] were grown in RPMI supplemented with 10% FBS and 5% penicillin/Streptomycin antibiotic at 37 °C in a humidified atmosphere containing 5% CO_2_ [48]. OMM1 (GNA11^Q209L^) was grown in Gibco DMEM supplemented with 10% FBC, 5% penicillin/Streptomycin, 1% Sodium pyruvate 100 mM, 1% MEM essential vitamin mixture and 1% NEAA mixture and 1% HEPES Buffer solution. No mutation is reported for BAP1, SF3B1, or EIF1AX in these cell lines. MP46 (GNAQ^Q209L^) and MP65 (GNA11^Q209L^) were grown in RPMI supplemented with 10% FBS and 5% penicillin/Streptomycin antibiotic at 37 °C in a humidified atmosphere containing 5% CO_2_. MP46 and MP65 are BAP1-deficient, no mutation for SF3B1 or EIF1AX in these cell lines are reported. Melanocytes were isolated from the healthy part of the choroid of two donor eyeballs (NHCM#1 and NHCM#2). Cell lines are regularly tested for mycoplasma and are mycoplasma-free. They were authenticated through short tandem repeat (STR) profiling.

### Biochemicals

Mithramycin A was from Santa Cruz (sc-200909) and SETDB1i was from MedChem Express (HY-141539).

### Pooled CRISPR library details

The custom oligonucleotide library with sgRNAs targeting ~140 human chromatin regulators genes (3–4 sgRNAs per target) was used as previously described [16]. To ensure library diversity, colonies were collected from 15 bacterial plates after transformation of 10-beta electrocompetent (New England Biolabs). The pool of plasmids was prepared for infection using an endotoxin-free Maxi prep kit (Qiagen).

### CRISPR-Cas9 screen

Human OMM1.3 uveal melanoma cells were first infected with the lentiCas9-Hygro (Addgene # 104995) and selected with hygromycin (200 μg/mL). Cells were then infected with the sgRNA library at a low MOI (< 1) to ensure a single sgRNA vector per cell. After 4 days of infection, cells were analyzed by flow cytometry and <20% of cells were EGFP-positive, corresponding to single vector copy. EGFP-positive cells were expanded for 10 days. A fraction of cells was collected at day 0 to ensure a proper coverage of sgRNAs. Medium was changed every 3 days. At day 35, cells from all conditions were collected and genomic DNA was extracted. Since melanin pigment may interfere with DNA-and/or RNA-based molecular profiling [49], we purified the samples using the OneStepTM PCR inhibitor Removal Kit (Zymo Research). The integrated sgRNAs were then amplified by PCR with primers containing multiplexing barcodes and adaptors and sequenced on the Illumina NextSeq500. Hits were selected based on the log2 fold change of sgRNA reads at day 35. Analyses and plots of the sequencing data were conducted using Prism 6 software (GraphPad Software). Data were analysed using the software Mageck, which calculates a score based on a fold change where either sgRNA is depleted or enriched compared to the control condition.

### mRNA preparation and real-time/quantitative PCR

The mRNAs were prepared using TRIzol (Fisher Scientific,15596026 T) according to a standard procedure. RT-qPCR was performed using SYBR® Green I (Fisher Scientific, 4368708) and Multiscribe Reverse Transcriptase (Applied Biosystems) and subsequently monitored using the ABI Prism 7900 Sequence Detection System (Applied Biosystems, Foster City, CA) as previously reported [50]. The detection of the RPLP0 mRMA was used to normalize the results. Primer sequences for each cDNA were designed using either Primer bank (https://pga.mgh.harvard.edu/primerbank/). Sequences are available upon request.

### RNA-sequencing

Reads were preprocessed to remove adapter and low-quality sequences (Phred quality score below 20). After this preprocessing, reads shorter than 40 bases were discarded for further analysis. These preprocessing steps were performed using cutadapt version 1.10. Reads were mapped to rRNA sequences using bowtie version 2.2.8 and reads mapping to rRNA sequences were removed for further analysis. Reads were mapped onto the hg38 assembly of Homo sapiens genome using STAR version 2.5.3a. Gene expression quantification was performed from uniquely aligned reads using htseq-count version 0.6.1p1, with annotations from Ensembl version 99 and “union” mode. Only non-ambiguously assigned reads have been retained for further analyses. Read counts have been normalized across samples with the median-of-ratios method [51]. Differential gene expression analysis was performed using the methodology implemented in the Bioconductor package DESeq2 version 1.16.1 [52]. *P*-values were adjusted for multiple testing by the method proposed by Benjamini and Hochberg [53]. Deregulated genes were defined as genes with log2(foldchange) ≥ 0.65 or ≤ −0.65 and adjusted *P*-value ≤ 0.05.

### Transient transfection of siRNA and infection of shRNA

Briefly, a single pulse of 50 nM of control siRNA, siRNA to SETDB1 (Sigma SASI_Hs02_00344324) or siRNA to CDC6 (Sigma SASI_Hs01_00047246 and SASI_Hs01_00047247) was administered to the cells at 50% confluency through transfection with 5 µl of Lipofectamine^TM^ RNAiMAX in Opti-MEM medium (Invitrogen, San Diego, CA, USA) as described [54].

### Cell cycle analysis

The Click-iT Plus EdU Alexa Fluor 647 Flow Cytometry Assay Kit (Invitrogen C10634) was used for detection of replicating OMM1.3 cells based on incorporation of 2 μM EdU (5-ethynyl 2′-deoxyuridine) into newly synthesized DNA for 2 h followed by its recognition with azide dyes via copper-mediated “click” reaction, according to the manufacturer’s protocol. DAPI and EdU (C10634, Invitrogen) double staining was used to measure DNA content in live cells by flow cytometry.

### Whole cell protein extractions and chromatin fractionation

Cells were washed with PBS and lysed on ice for 5 min in NP40 buffer (50 mM Tris pH 7.5, 1% NP40, 150 mM NaCl, 10% Glycerol, 1 mM EDTA) supplemented with protease and phosphatase inhibitors (Roche). Lysates were centrifuged at 15,000 rpm for 15 min and the protein concentration was quantified using BCA (Pierce). Chromatin fractionation performed as described [55]. All lysates were freshly prepared and supplemented with Laemmli loading buffer with subsequent boiling for immunoblotting.

### Immunoblot assays

Briefly, cell lysates (30 µg) were separated using SDS-PAGE, transferred onto a PVDF membrane as previously described [56] and subsequently exposed to the appropriate antibodies, anti-SETDB1 (VMA00243; 1/1000) from Biorad, anti-p21 (2947; 1/1000), anti-CDC6 (3387; 1/1000), anti-phospho CHEK2 (2197; 1/1000), anti-CHEK2 (6334; 1/1000), and anti-PARP (9542, 1/1000), from Ozyme, anti MCM6 (ab201683; 1/1000) from Abcam, anti-β-ACTIN (sc-47778; 1/1000), anti-GAPDH (sc-47724; 1/1000) and anti-HSP90 (sc-13119; 1/1000) from Santa Cruz Biotechnology, H3K9me2/3 (5327; 1/1000) from Cell Signaling Technology. The proteins were visualized using the ECL system (Amersham). The immunoblots shown are representative of at least 3 independent experiments.

### Cell growth assay

Human uveal melanoma cells were seeded onto six-well plates at low density, allowed to adhere overnight and cultured as indicated. Then, the colonies were stained with 0.04% crystal violet/2% ethanol in PBS for 30 min. Photographs of the stained colonies were captured. Crystal violet was then solubilized and growth was monitored by measuring the absorbance at 561 nm as previously reported [57]. Photographs of the stained colonies were captured. The assay was performed in triplicate.

### Immunofluorescence staining

Immunofluorescence experiments were carried out as previously described [58]. Briefly, cells grown on glass coverslips were fixed in 4% formaldehyde in PBS supplemented with 0.1% Triton ×-100 for 15 min at room temperature prior to permeabilization in 0.1% Triton ×-100 for 10 min. After 1 h of blocking with 1% BSA in PBS containing 0.1% Tween 20, the cells were stained overnight at 4 °C in a humidified chamber in a blocking solution with antibodies to γ-H2AX (1/500, Abcam ab11174), 53BP1 (1/50, Bethyl, IHC-00001). Primary antibody detection was achieved via incubation with anti-rabbit or anti-mouse Alexa Fluor 594- or 488-conjugated secondary antibodies (Invitrogen) for 90 min at room temperature. The slides were mounted in DAKO mounting medium supplemented with Hoechst (1/1000, Invitrogen, #H3570) and examined using a 40× oil immersion objective with a NIKON AR1 confocal microscope. Representative experiments are shown.

### Animal experimentation

Animal experiments were performed in accordance with French law and approved by a local institutional ethical committee. The animals were maintained on a 12-h light/dark cycle in a temperature-controlled facility at 22 °C and provided free access to food (standard laboratory chow diet). Human OMM1 melanoma cells (5 × 10^6^ cells) were subcutaneously inoculated into 8-wk-old male and female immune-deficient Nod scid gamma (NSG) mice (Janvier Laboratory). When the tumors became palpable, mice received intraperitoneal injection of Mithramycin (1 mg/kg) 3 times per week dissolved in phosphate buffered saline (PBS). Overall, Mithramycin A was well tolerated, with only transient weight loss in some mice that resolved after treatment discontinuation for one or two rounds. Control mice were injected with PBS alone. The tumor growth curves were determined after measuring the tumor volume using the equation V = (L × W2)/2 as previously reported [59]. Mice were randomly assigned to the different treatment groups.

### Statistics

No data were excluded from the analyses. Investigators were not blinded. No statistical methods were used to determine the sample size. Sample size was determined to be adequate based on the magnitude and consistency of measurable differences between groups. The statistical analyses were performed by GraphPad Prism 6 (GraphPad Software). Statistical significance between groups was determined using GraphPad Prism as indicated in the legends. *p-value ≤ 0.05; **p-value ≤ 0.01; ***p-value ≤ 0.001; ****p-value ≤ 0.0001.

## Supplementary information

Supplementary Figures_Legends

Original blots

## Data Availability

The RNA-sequencing data generated and/or analyzed during this study have been deposited in the NCBI Gene Expression Omnibus (GEO) database (https://www.ncbi.nlm.nih.gov/geo/) under the SuperSeries GSE302422.
