# Supplementary material for: SETDB1 is critically required for uveal melanoma growth and represents a promising therapeutic target
Source: Cell Death Dis. 2025 Oct 24;16(1):754. doi: 10.1038/s41419-025-08084-z (PMC12552495; doi:10.1038/s41419-025-08084-z)
Supplement: Supplementary file 1 — Supplementary Figures_Legends [file 41419_2025_8084_MOESM1_ESM.pdf]

Krossa_Supplementary figure 1

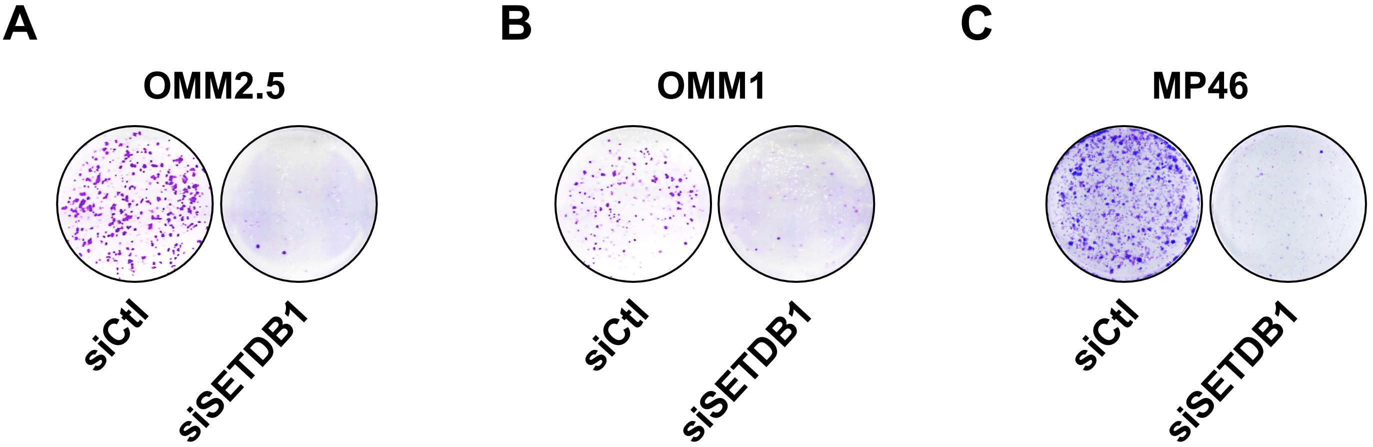

Krossa_Supplementary figure 2

A

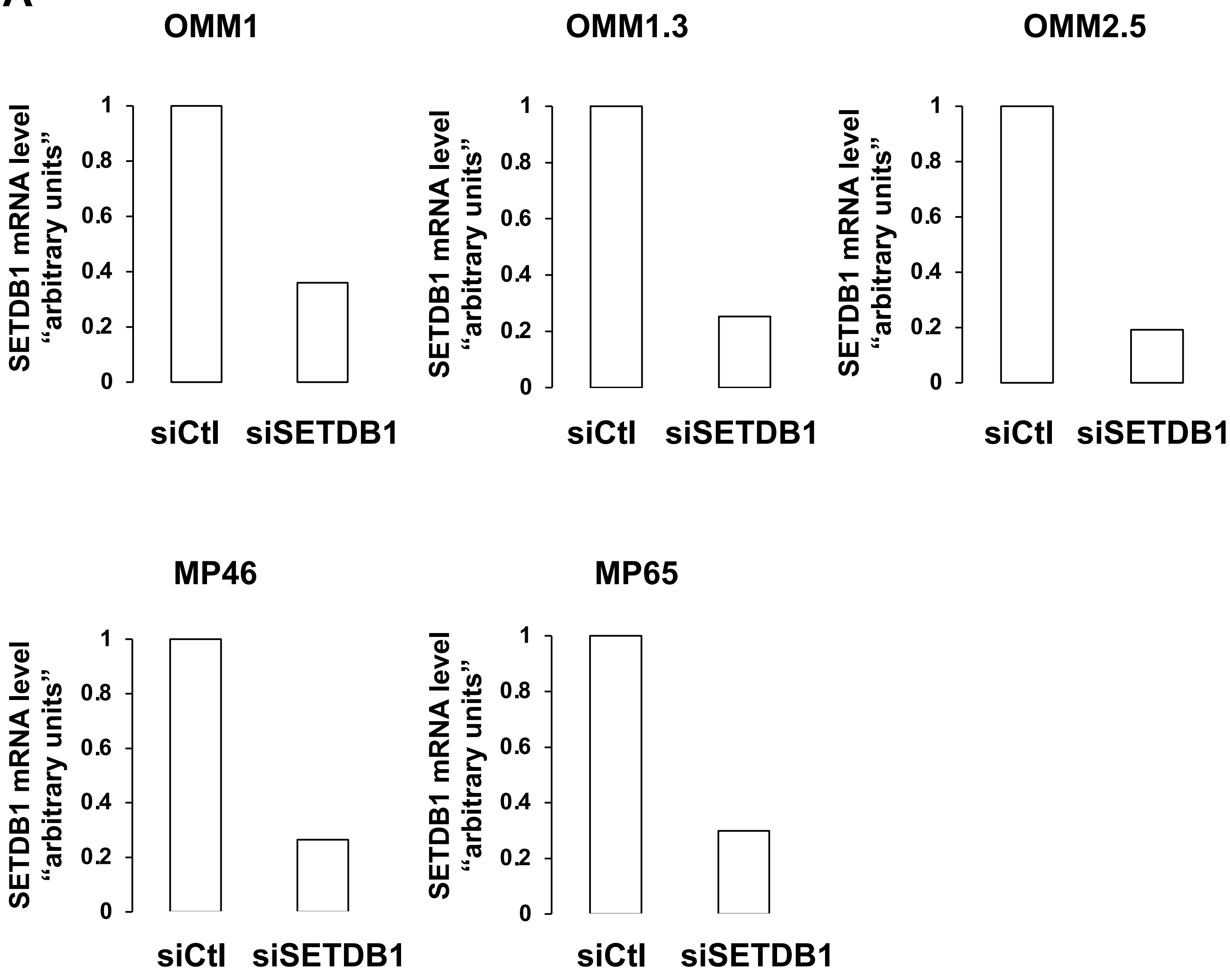

B

GSEA (Gene set enrichment analysis) of downregulated genes

	Biological process with the highest enrichment score	FDR-qval	FWER p-val
1	GOBP NULEOSIDE MONOPHOSPHATE BIOSYNTHETIC PROCESS	0.049	0.023
2	GOBP TELOMERE MAINTENANCE	0.030	0.028
3	GOBP POSITIVE REGULATION OF DNA REPLICATION	0.027	0.030
4	GOBP DNA REPLICATION INITIATION	0.034	0.035
5	GOBP DEPENDENT DNA REPLICATION	0.029	0.035
6	GOBP REGULATION OF TELOMERE MAINTENANCE VIA TELOMERE LENGTHENING	0.027	0.036
7	GOBP DNA STRAND ELONGATION	0.026	0.036
8	GOBP MATURATION OF 5 8S RRNA	0.023	0.036
9	GOBP REGULATION OF DNA REPLICATION	0.023	0.043
10	GOBP INTERSTRAND CROSS LINK REPAIR	0,0215	0,049

Krossa_Supplementary figure 3

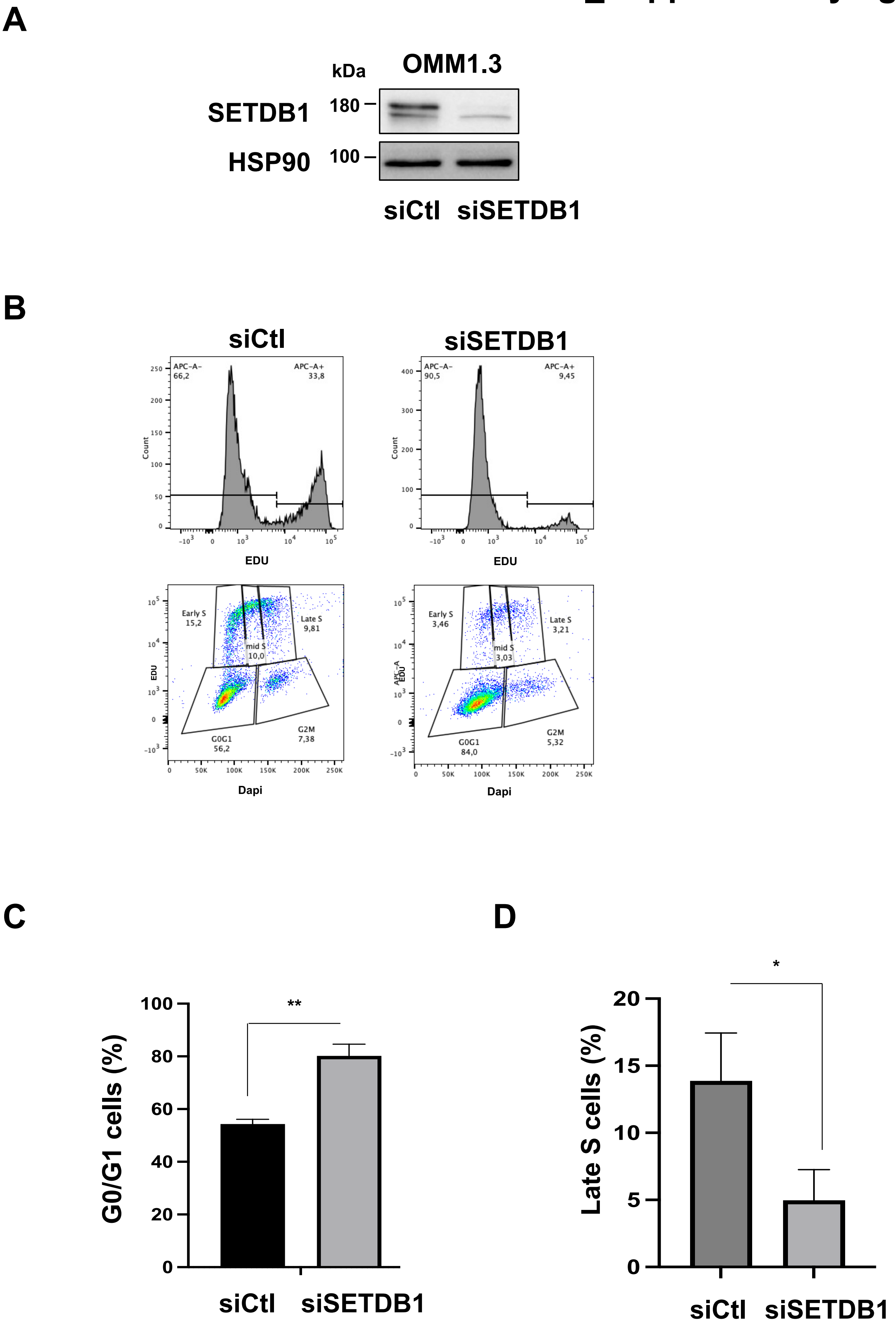

Krossa_Supplementary figure 4

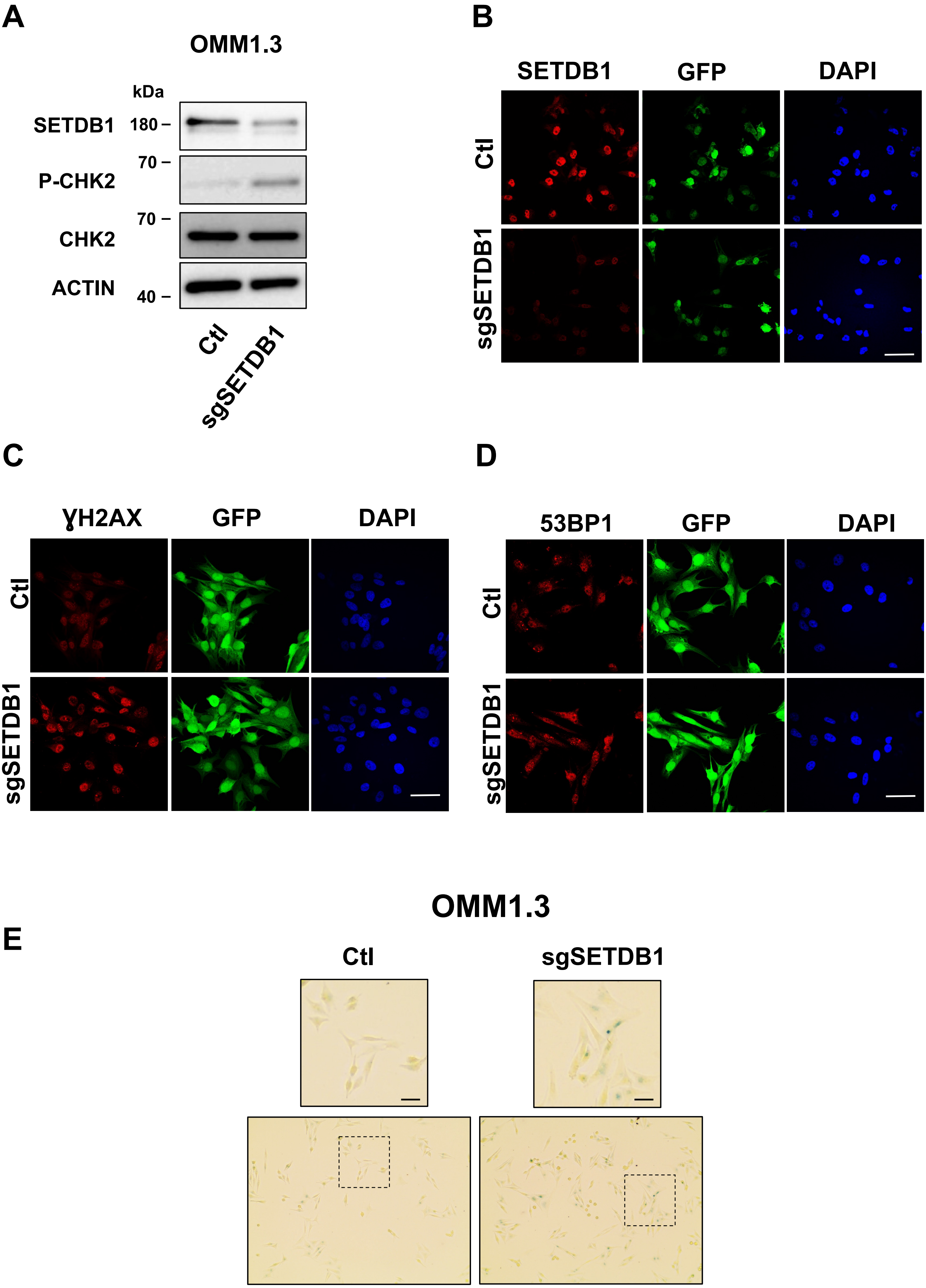

Krossa_Supplementary figure 5

A

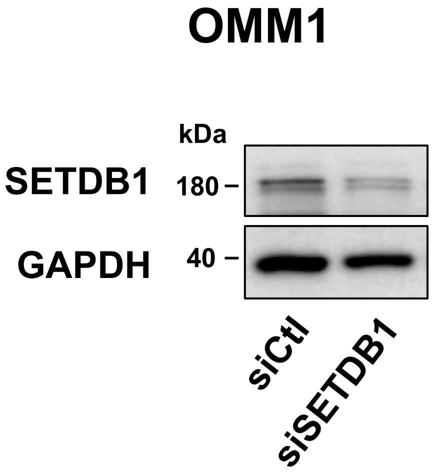

B

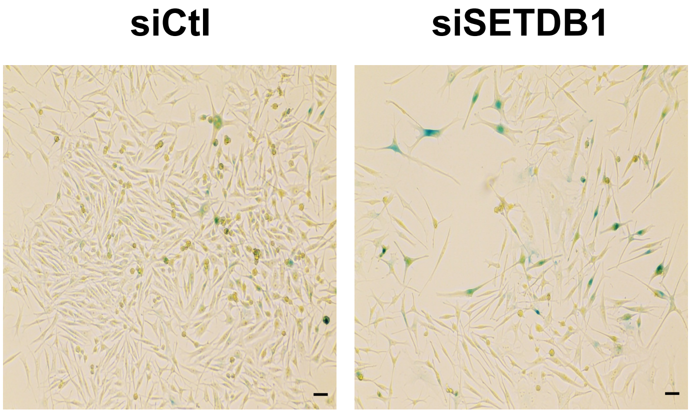

Krossa_Supplementary figure 6

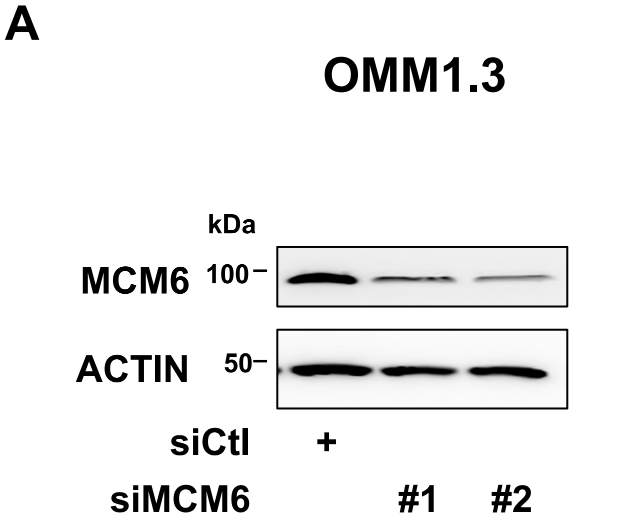

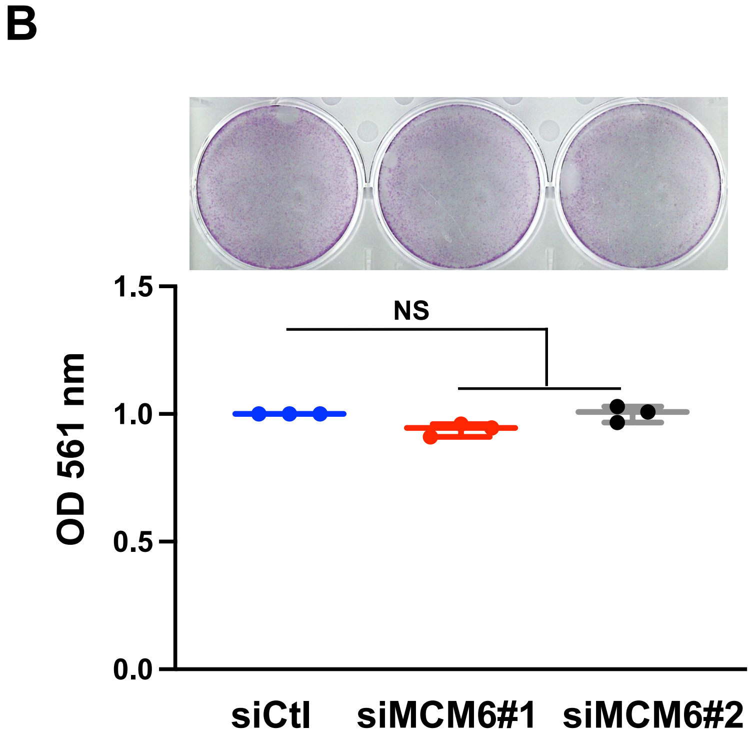

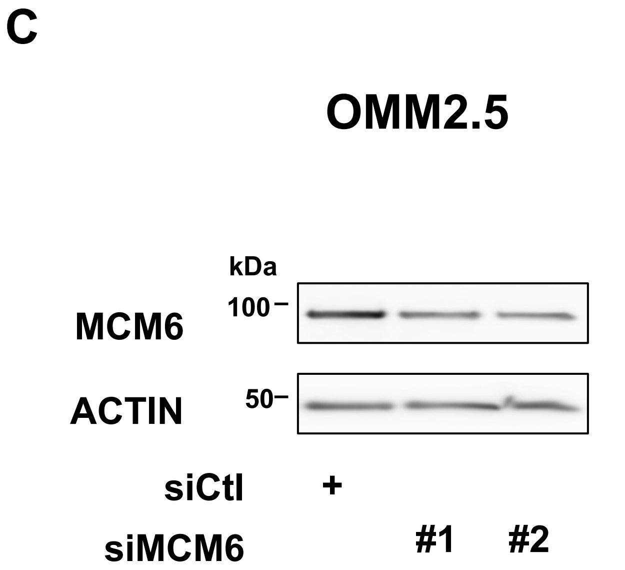

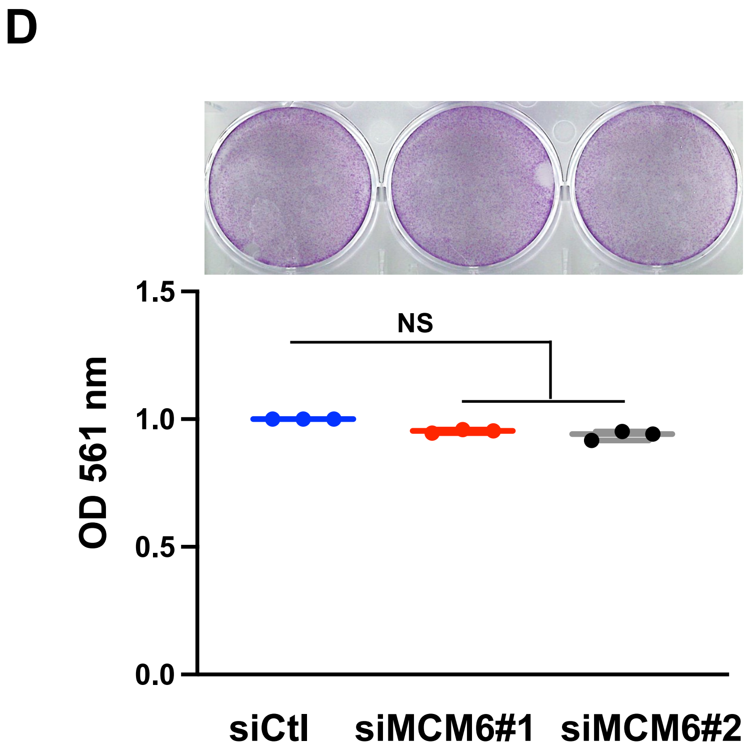

Krossa_Supplementary figure 7

A

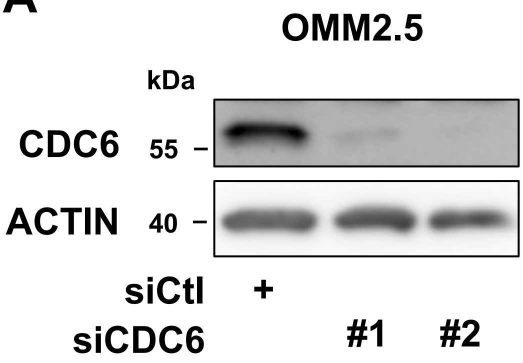

B

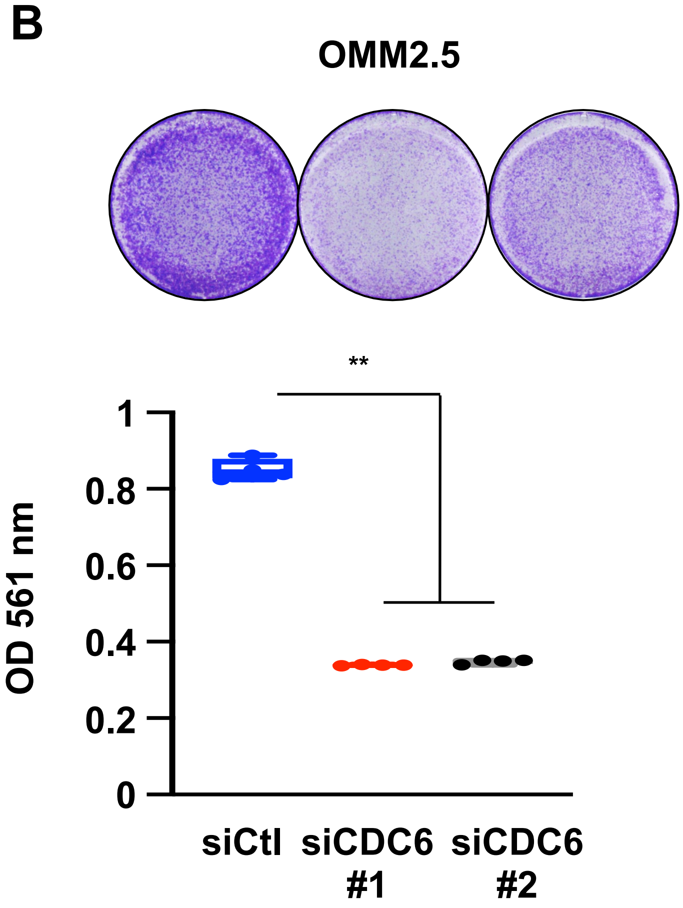

Krossa_Supplementary figure 8

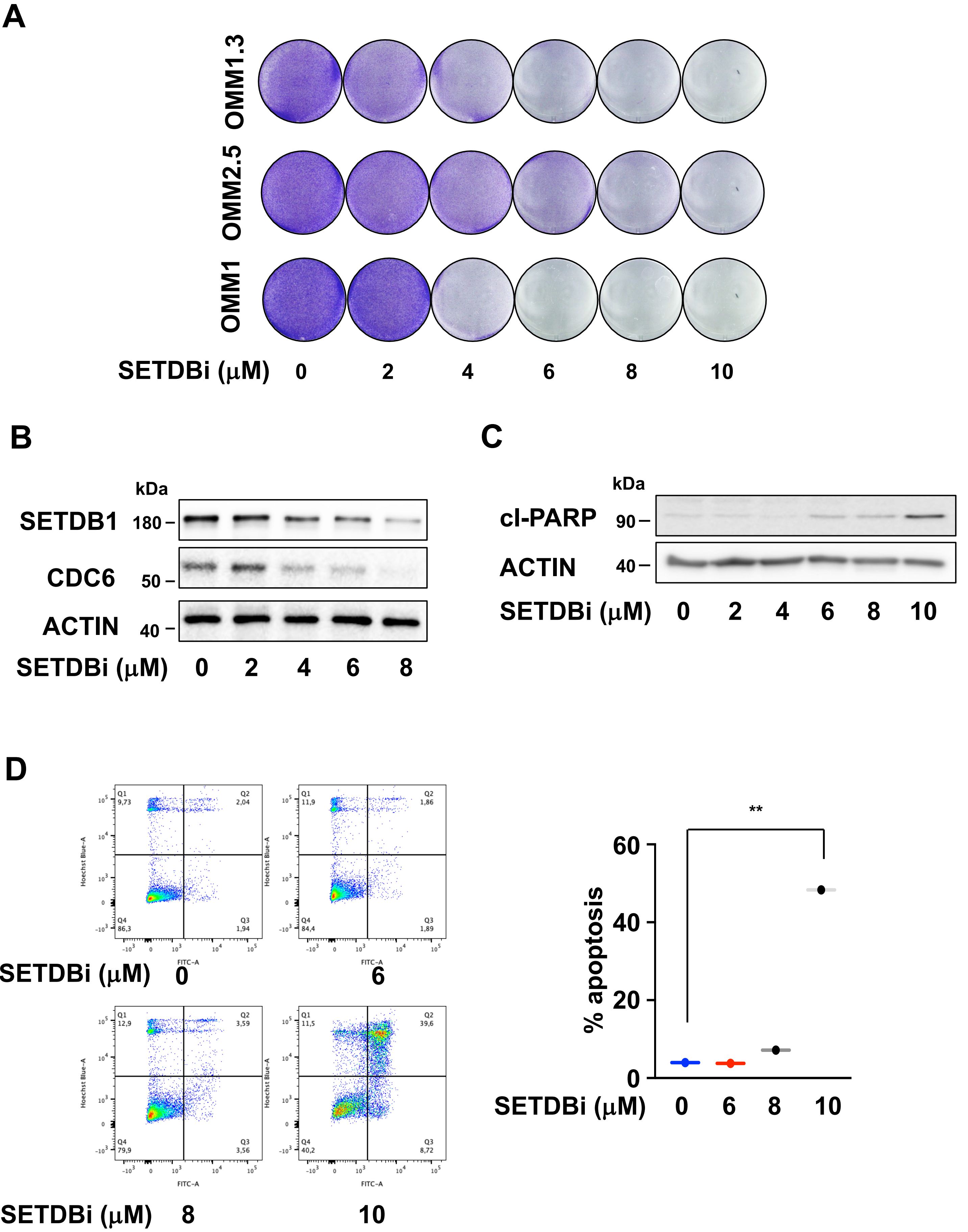

Krossa_Supplementary figure 9

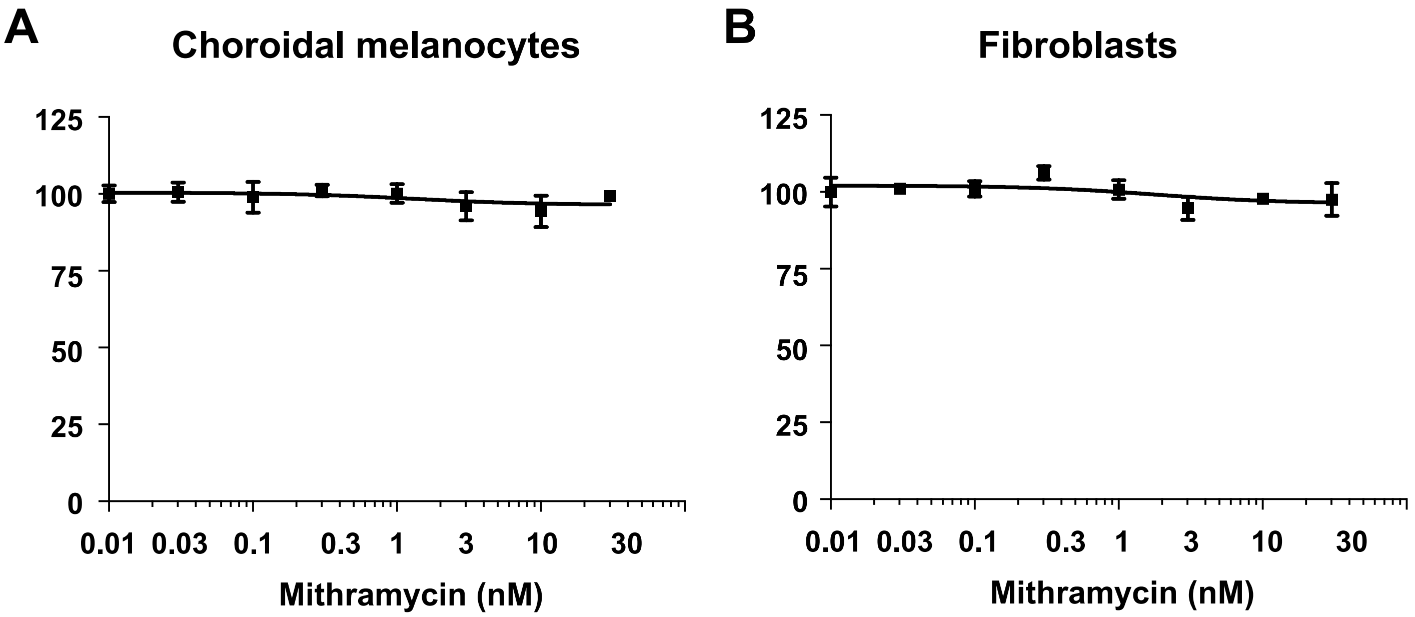

Supplementary figures

Supplementary figure 1: SETDB1 plays a key role in uveal melanoma cell growth. (A-C) Cell growth capacity of metastatic (OMM2.5 and OMM1) and primary (MP46) uveal melanoma cells treated with a control siRNA (Ctl) or with an siRNA for SETDB1 (siSETDB1).

Supplementary figure 2: SETDB1 regulates uveal melanoma cell replication. (A) SETDB1 mRNA levels of human OMM1, OMM1.3, OMM2.5 metastatic uveal melanoma cells and of human MP46, MP65 primary uveal melanoma cells treated with a control siRNA (siCtl) or an siRNA to SETDB1 for 48 hrs were analysed by RT-qPCR to validate SETDB1 inhibition in the transcriptomic profiling. **(B)** Top ten significantly deregulated GO terms in SETDB1 knockdown versus control condition. Four GO terms are related to replication.

Supplementary figure 3: SETDB1 knockdown delays S phase.
(A) Immunoblot to SETDB1 in lysates of OMM1.3 cells treated with control siRNA or an siRNA to SETDB1 for 96 hrs. HSP90 is used as a loading control. **(B)** DNA content was measured in live cells treated as in A using DAPI/EdU double staining and flow cytometry. **(C-D)** G0/G1 and late S are shown. n=3. Data are mean ± SD; **p=0.0085, *p=0.0212 two-way ANOVA.

Supplementary figure 4: SETDB1 knockdown induces a DNA damage response.
(A) Immunoblot of whole-cell lysates of OMM1.3 infected with control sgRNA (Ctl) or a sgRNA to SETDB1 with the indicated antibodies. β-Actin was used as a loading control. **(B)** Control and SETDB1 KD OMM1.3 cells were analysed by immunofluorescence for SETDB1. Cell nuclei were counterstained with DAPI. Representative fluorescence images are shown. Bar=20µM. **(C)** Cells treated as in A were analysed by immunofluorescence for H2AX phosphorylated on Ser139 (γH2AX). Cell nuclei were counterstained with DAPI. Representative fluorescence images are shown. Bar=20µM. **(D)** Cells treated as in A were analysed by immunofluorescence for 53BP1. Cell nuclei were counterstained with DAPI. Representative fluorescence images are shown. Bar=20µM. **(E)** SA-β-Gal staining of OMM1.3 infected with control sgRNA (Ctl) or a sgRNA to SETDB1.

Supplementary figure 5: SETDB1 knockdown induces a senescence-like state. (A) Immunoblot to SETDB1 of OMM1 cells treated with control siRNA or siRNA to SETDB1 (siSETDB1) for 96 hrs. GAPDH is used as a loading control. **(B)** SA-β-Gal staining of OMM1 cells treated with control siRNA or siRNA to SETDB1 for 96 hrs.

Supplementary figure 6: MCM6 knockdown does not affect metastatic uveal melanoma cell proliferation. (A) Western blot of OMM1.3 cells treated with a control siRNA or with two different siRNA to MCM6 for 72 hrs. β-Actin was used as a loading control. **(B)** Proliferation assay of OMM1.3 treated with a control siRNA (siCtl) or siRNA to MCM6 grown for 72 hrs. **(C)** Western blot of OMM2.5 cells treated with a control siRNA or with two different siRNA to MCM6 for 72 hrs. β-Actin was used as a loading control. **(D)** Proliferation assay of OMM2.5 treated with a control siRNA (siCtl) or siRNA to MCM6 grown for 72 hrs.

Supplementary figure 7: CDC6 is required for proliferation of metastatic uveal melanoma cells. (A) Immunoblot to CDC6 of OMM2.5 cells treated with a control siRNA (Ctl) or with two different siRNA to CDC6. β-Actin was used as a loading control. **(B)** OMM2.5 cells treated with a control siRNA (siCtl) or siRNAs to CDC6 were seeded at the same density and cultured for 10 days (top), crystal violet quantification at OD 561 nm (bottom). Mann-Whitney test was performed for comparison between groups, n=4. Data are the mean ± SEM. **p=0.0286.

Supplementary figure 8: Anti-SETDB1 therapy reduces cell proliferation and viability of uveal melanoma cells. (A) OMM1.3, OMM2.5, OMM1 metastatic uveal melanoma cells were seeded at low density and cultured for 10 days in absence or presence of increasing concentration of SETDB1 inhibitor (SETDB1i). Representative images of three independent experiments are shown. **(B)** Immunoblot to SETDB1 and CDC6 in control OMM1.3 cells and OMM1.3 cells treated with SETDB1i. β-Actin was used as a loading control. **(C)** Immunoblot analysis of metastatic uveal melanoma cells exposed to SETDB1i for 72 hrs with the cleaved PARP (cl PARP) antibodies. β-Actin was used as a loading control. **(D)** Analysis of apoptosis in control OMM1.3 cells and OMM1.3 cells treated with SETDB1i at the indicated concentrations for 96 hrs.

Annexin V diagram and quantitation of the percentage of late apoptotic cells using the Annexin V assay, n=3. *p*-value was derived from Welch's t-test. **p=0.0022.

Supplementary figure 9: Mithramycin A effect on normal uveal melanocytes and fibroblasts. (A) Growth inhibition curve is shown for Mithramycin A at 96 h of treatment of normal uveal melanocytes isolated from the healthy part of the choroid of a donor eyeball (n = 3). Data are mean ± SEM. **(B)** Growth inhibition curve is shown for Mithramycin A at 96 h of treatment of fibroblasts (n = 3). Data are mean ± SEM.
