# Supplementary material for: SETDB1 is critically required for uveal melanoma growth and represents a promising therapeutic target
Source: Cell Death Dis. 2025 Oct 24;16(1):754. doi: 10.1038/s41419-025-08084-z (PMC12552495; doi:10.1038/s41419-025-08084-z)

Figure 1C: SETDB1

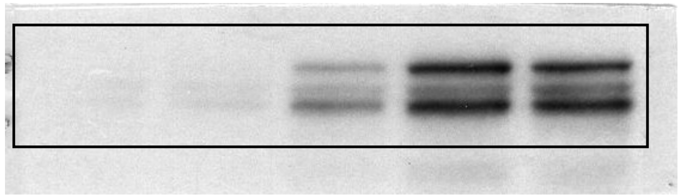

Figure 1C: HSP90

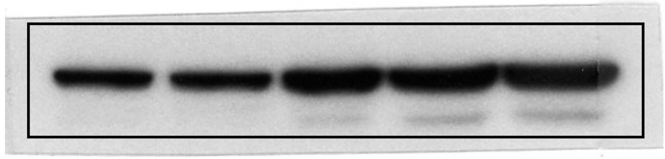

Figure 1D: SETDB1

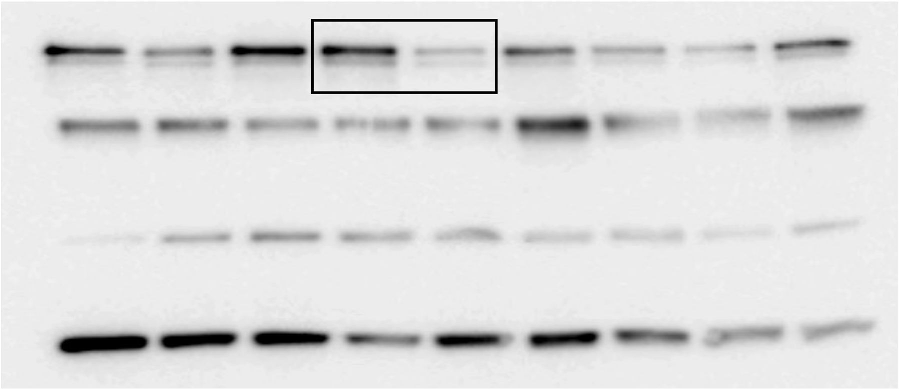

Figure 1D: ACTIN

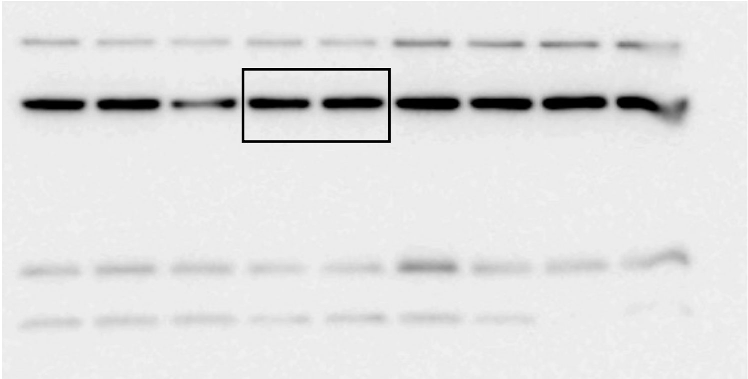

Figure 1G: SETDB1

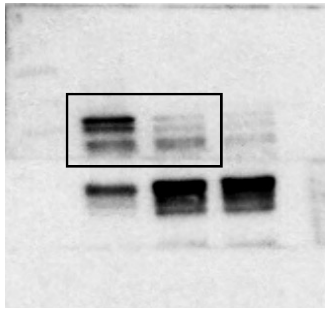

Figure 1G: ACTIN

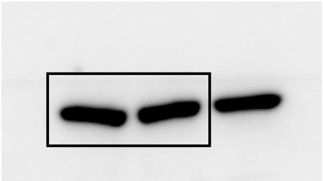

Figure 1I: SETDB1

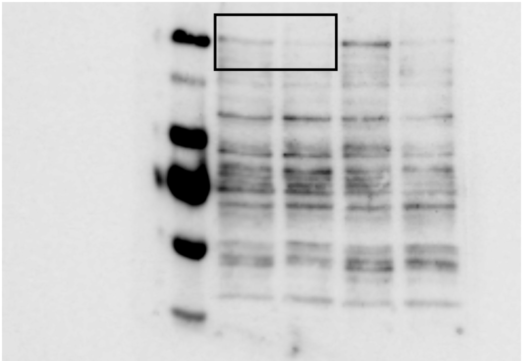

Figure 1I: H3K9me2/3

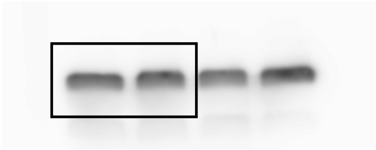

Figure 1I: Histones

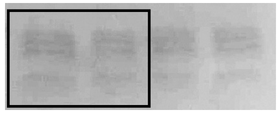

Figure 2F: SETDB1

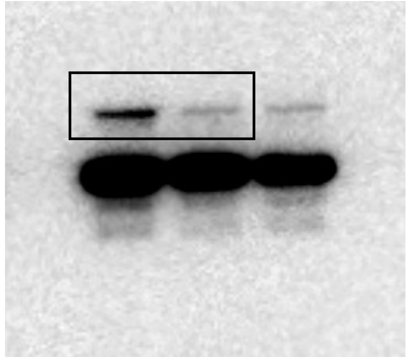

Figure 2F: ORC1

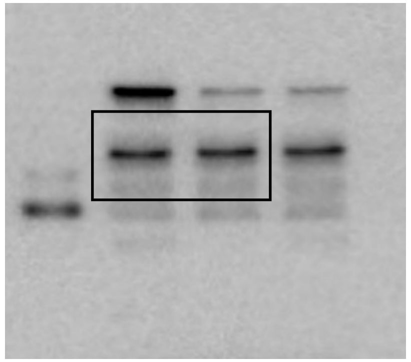

Figure 2F: CDC6

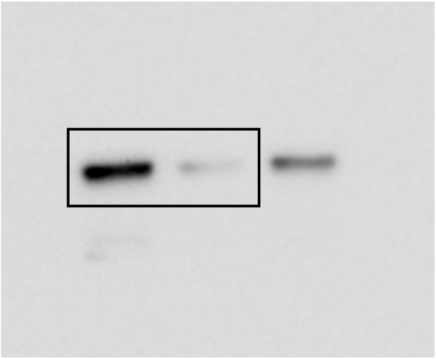

Figure 2F: MCM6

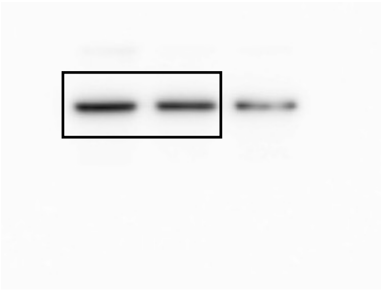

Figure 2F: MCM7

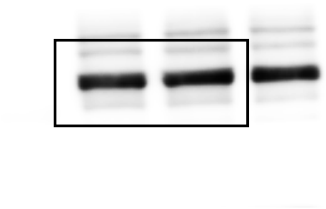

Figure 2F: GAPDH

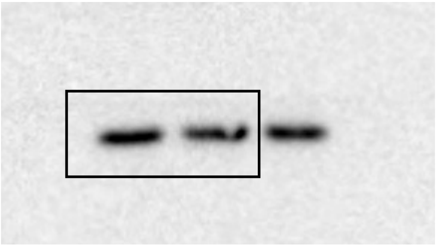

Figure 3A: SETDB1

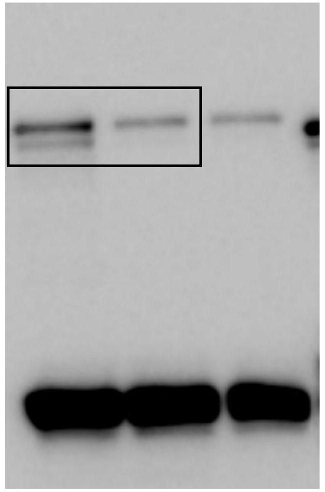

Figure 3A: P-CHK2

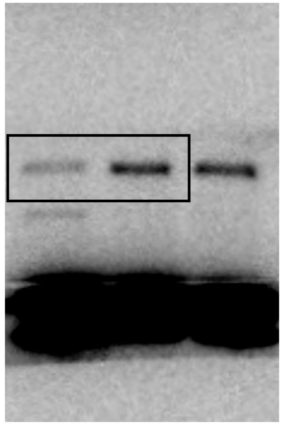

Figure 3A: CHK2

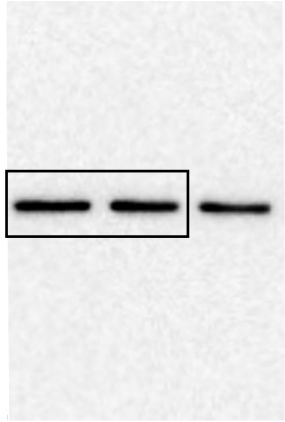

Figure 3A: ACTIN

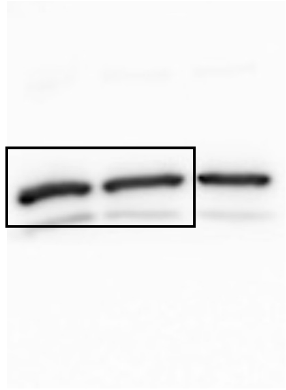

Figure 3E: SETDB1

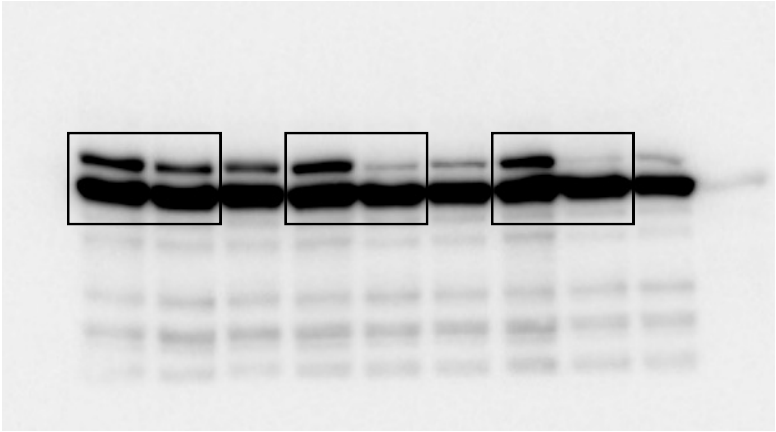

Figure 3E: P21

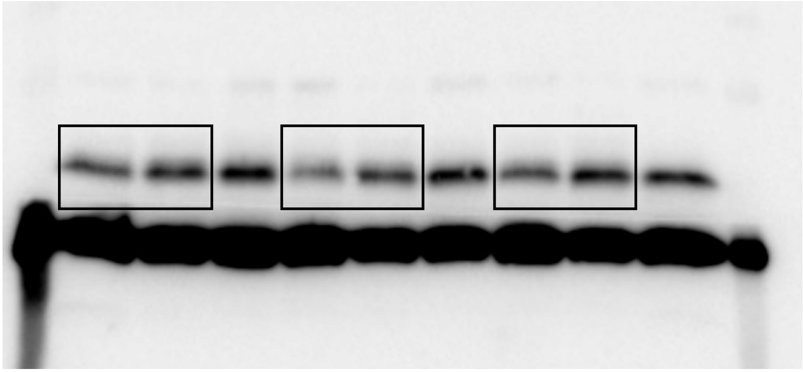

Figure 3E: ACTIN

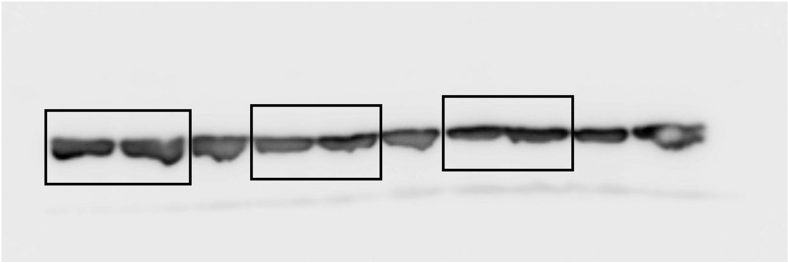

Figure 4A: SETDB1

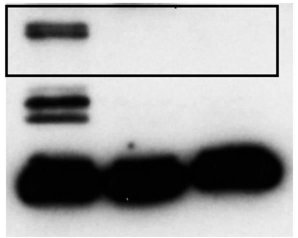

Figure 4A: P-CHK2

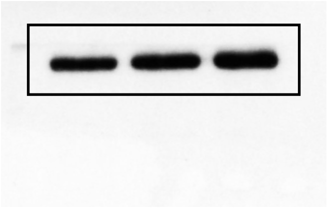

Figure 4A: CHK2

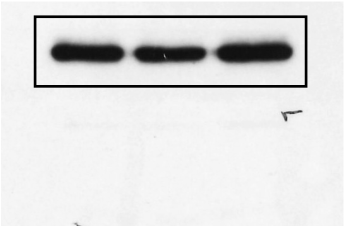

Figure 4A: HSP90

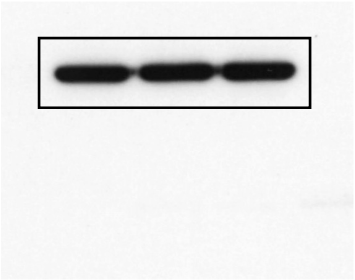

Figure 5B: SETDB1

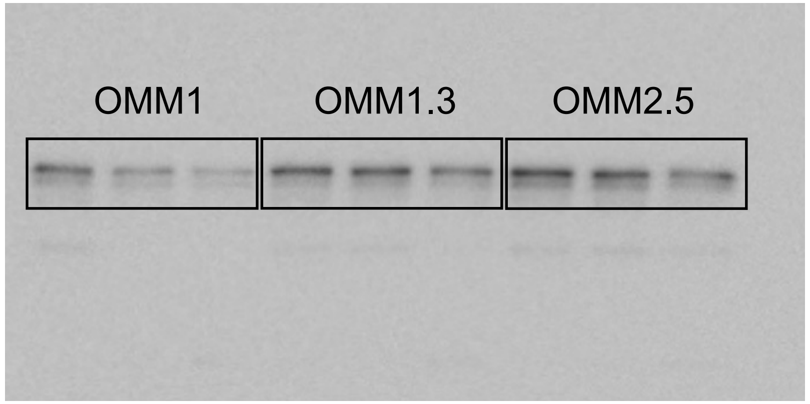

Figure 5B: CDC6

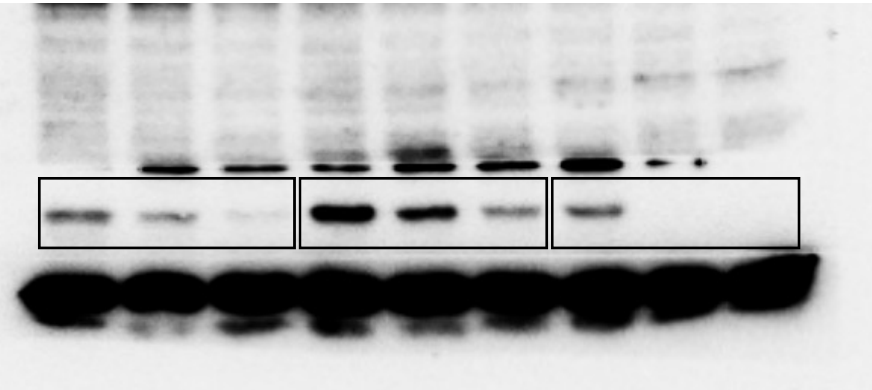

Figure 5B: HSP90

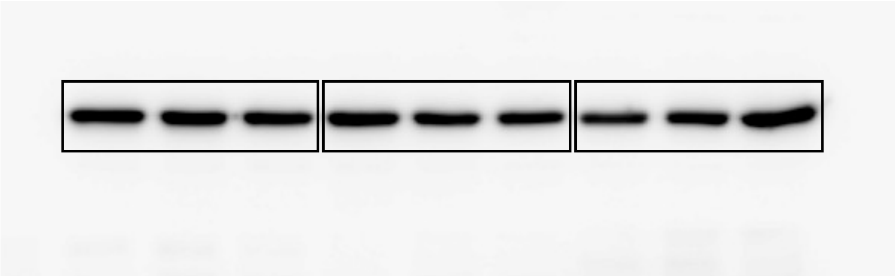

Figure 5C: clPARP

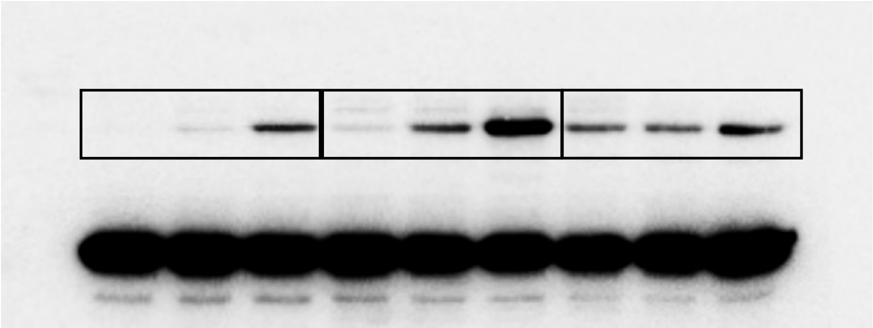

Figure 5C: ACTIN

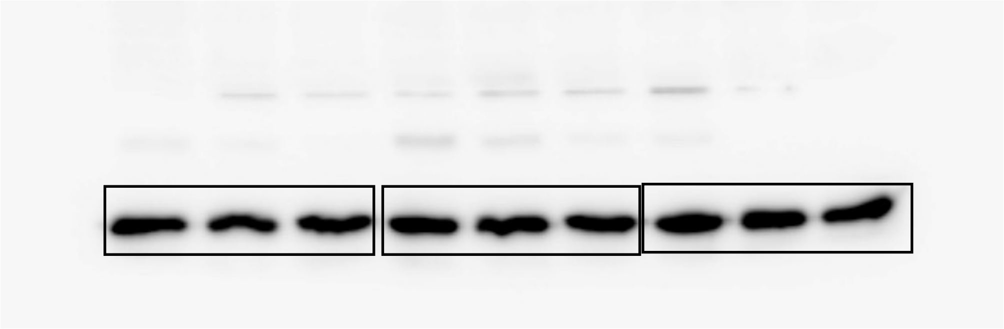

Supplementary figure 3A: SETDB1

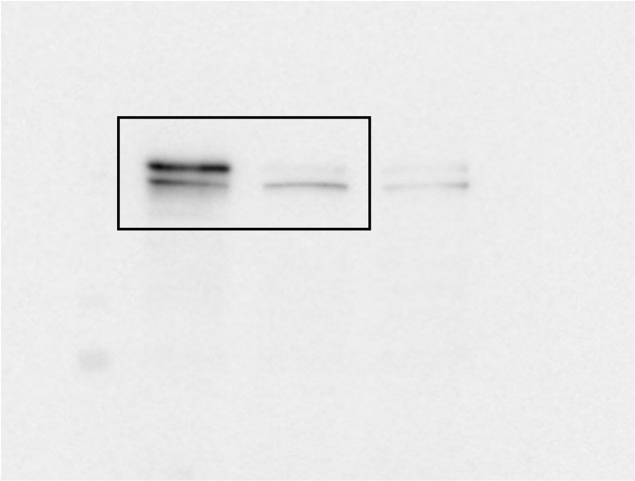

Supplementary figure 3A: HSP90

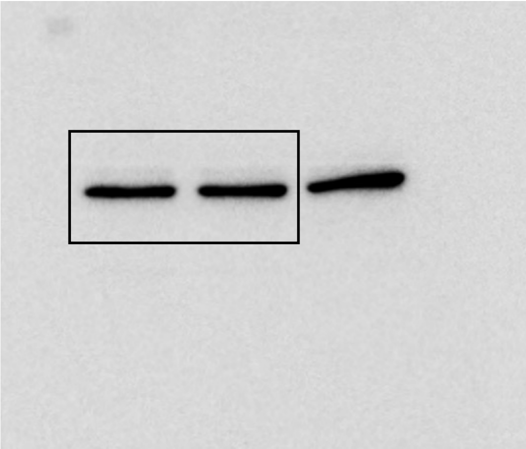

Supplementary figure 4A: SETDB1

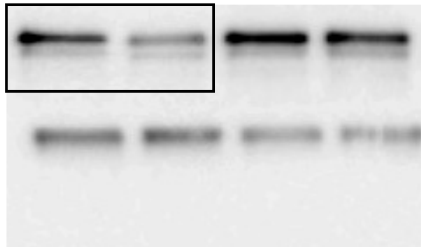

Supplementary figure 4A: P-CHK2

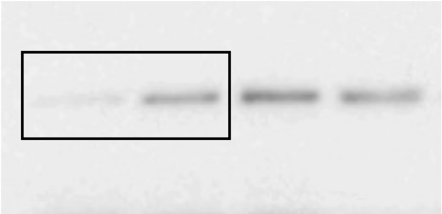

Supplementary figure 4A: CHK2

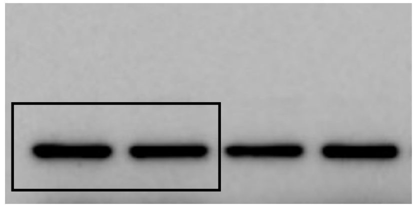

Supplementary figure 4A: ACTIN

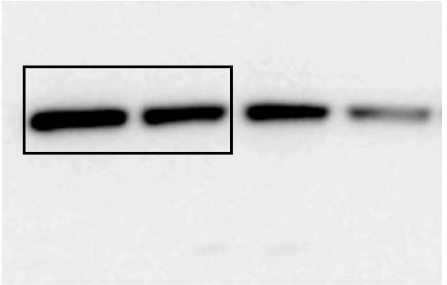

Supplementary figure 5: SETDB1

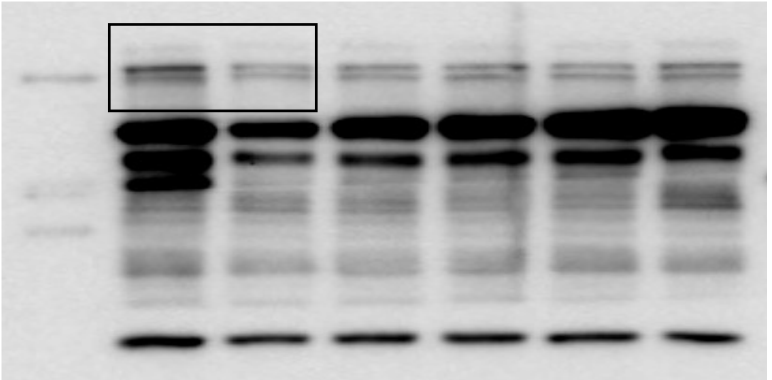

Supplementary figure 5: GAPDH

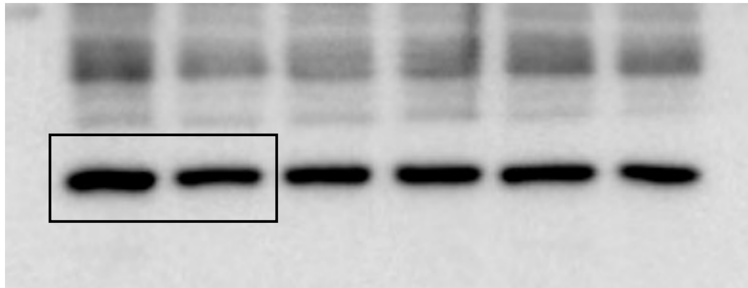

Supplementary figure 6A: MCM6

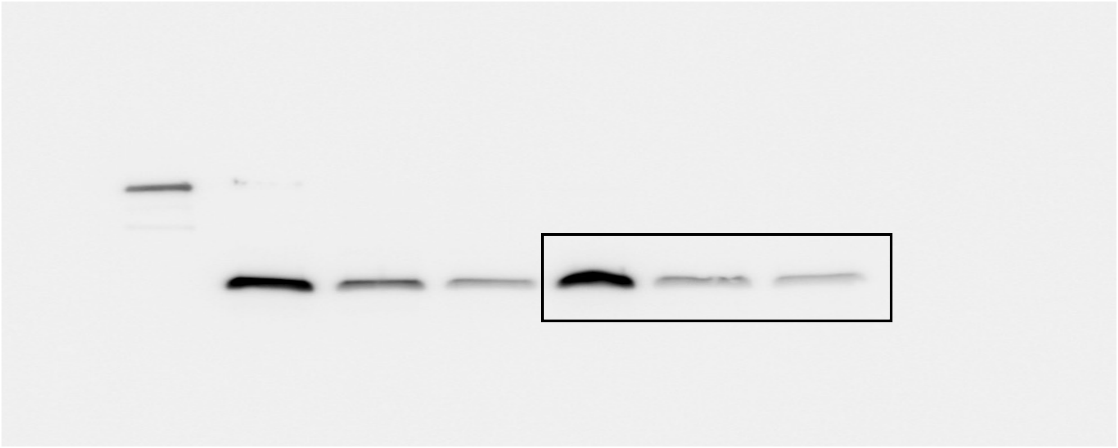

Supplementary figure 6C: MCM6

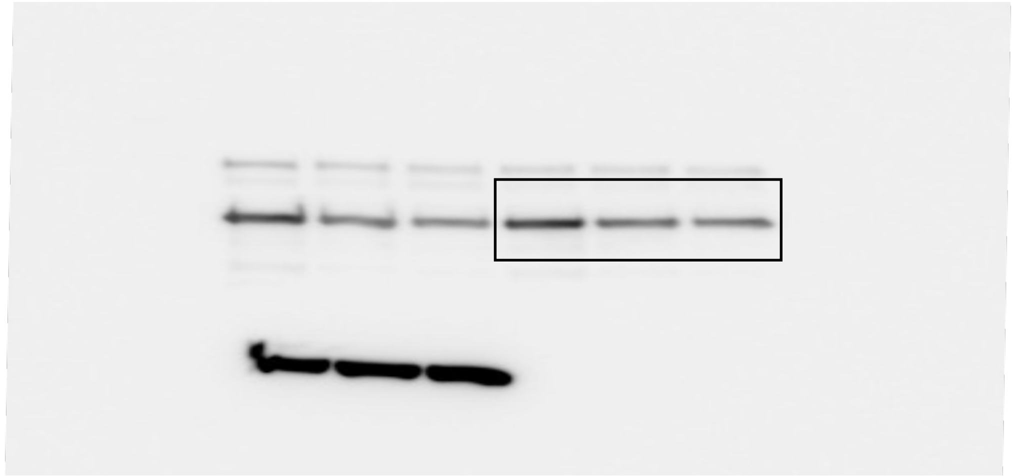

Supplementary figure 6A: ACTIN

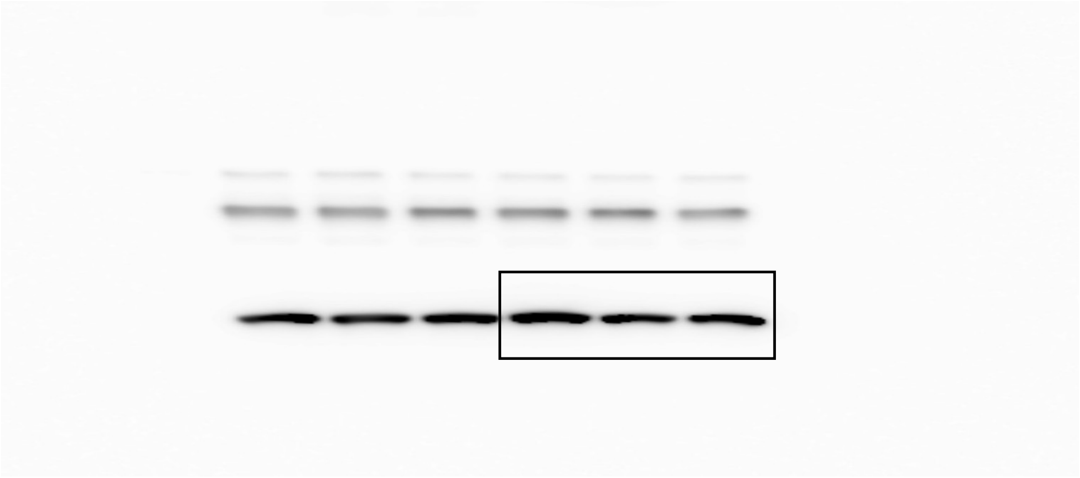

Supplementary figure 6C: ACTIN

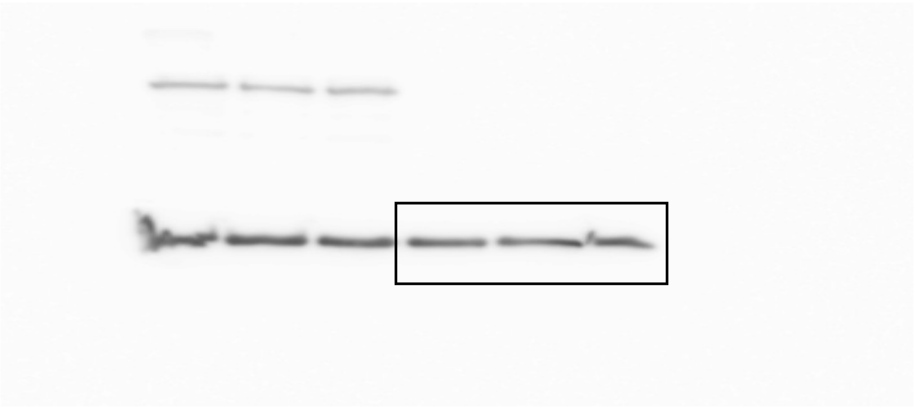

Supplementary figure 7A: CDC6

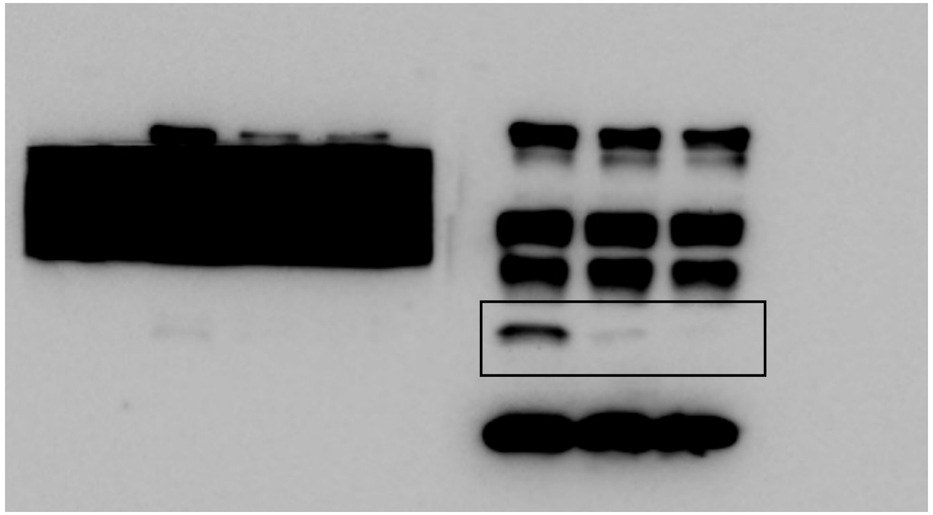

Supplementary figure 7A: ACTIN

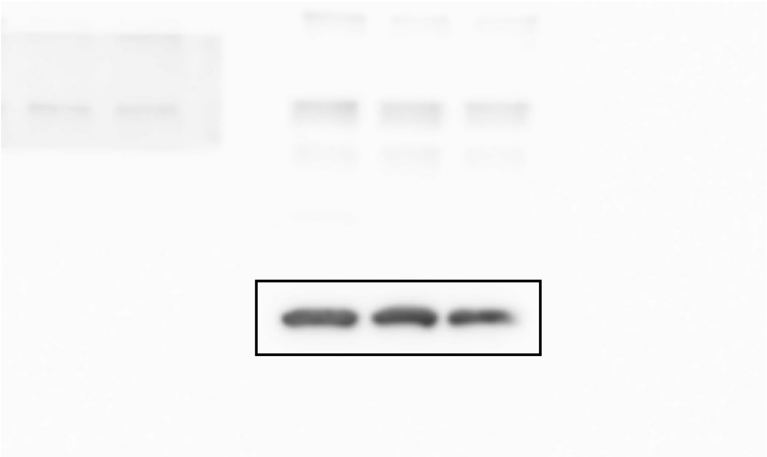

Supplementary figure 8B: SETDB1

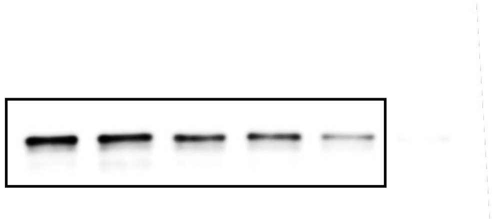

Supplementary figure 8B: CDC6

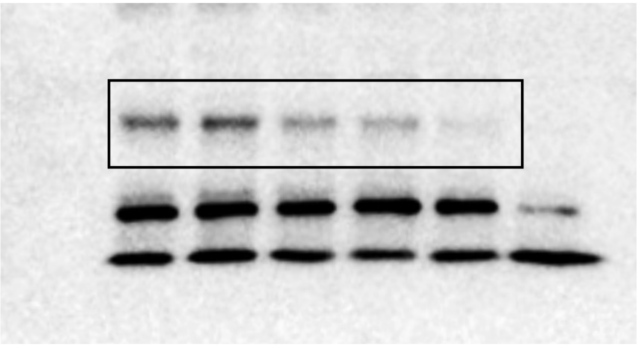

Supplementary figure 8B: ACTIN

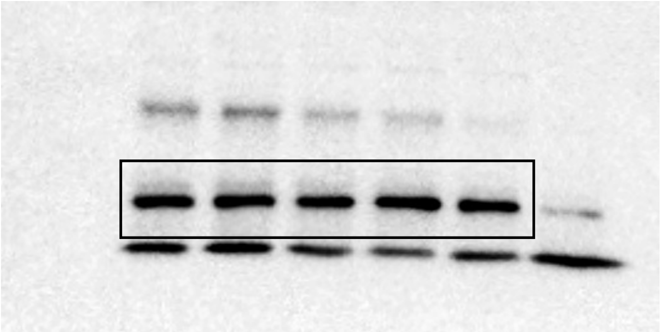

Supplementary figure 8C: clPARP

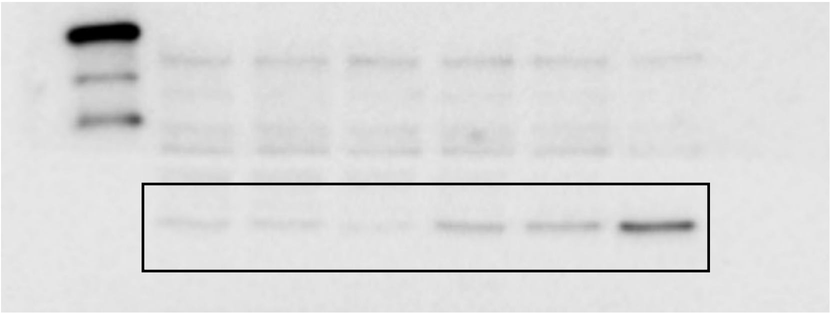

Supplementary figure 8C: ACTIN

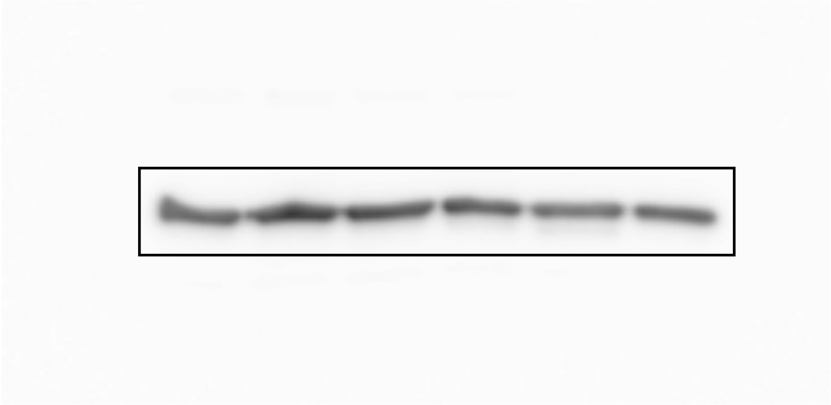

Supplement: Supplementary file 2 — Original blots [file 41419_2025_8084_MOESM2_ESM.pdf]
